# Superparamagnetic Multifunctionalized Chitosan Nanohybrids for Efficient Copper Adsorption: Comparative Performance, Stability, and Mechanism Insights

**DOI:** 10.3390/polym15051157

**Published:** 2023-02-24

**Authors:** Ahmed A. Al-Ghamdi, Ahmed A. Galhoum, Ahmed Alshahrie, Yusuf A. Al-Turki, Amal M. Al-Amri, S. Wageh

**Affiliations:** 1 Department of Physics, Faculty of Science, King Abdulaziz University, Jeddah 21589, Saudi Arabia; 2 Nuclear Materials Authority, El-Maadi, Cairo P.O. Box 530, Egypt; 3 Centre of Nanotechnology, King Abdulaziz University, Jeddah 21589, Saudi Arabia; 4 Department of Electrical and Computer Engineering, Faculty of Engineering, King Abdulaziz University, Jeddah 21589, Saudi Arabia; 5 K. A. CARE Energy Research and Innovation Center, King Abdulaziz University, Jeddah 21589, Saudi Arabia; 6 Physics Department, Rabigh College of Science and Arts, King Abdulaziz University, P.O. Box 344, Rabigh 21911, Saudi Arabia

**Keywords:** superparamagnetic nanohybrids, chitosan derivatives, copper adsorption, polyamine and amino acid moieties, selectivity, mathematical modeling

## Abstract

To limit the dangers posed by Cu(II) pollution, chitosan-nanohybrid derivatives were developed for selective and rapid copper adsorption. A magnetic chitosan nanohybrid (r-MCS) was obtained via the co-precipitation nucleation of ferroferric oxide (Fe_3_O_4_) co-stabilized within chitosan, followed by further multifunctionalization with amine (diethylenetriamine) and amino acid moieties (alanine, cysteine, and serine types) to give the TA-type, A-type, C-type, and S-type, respectively. The physiochemical characteristics of the as-prepared adsorbents were thoroughly elucidated. The superparamagnetic Fe_3_O_4_ nanoparticles were mono-dispersed spherical shapes with typical sizes (~8.5–14.7 nm). The adsorption properties toward Cu(II) were compared, and the interaction behaviors were explained with XPS and FTIR analysis. The saturation adsorption capacities (in mmol.Cu.g^−1^) have the following order: TA-type (3.29) > C-type (1.92) > S-type (1.75) > A-type(1.70) > r-MCS (0.99) at optimal pH_0_ 5.0. The adsorption was endothermic with fast kinetics (except TA-type was exothermic). Langmuir and pseudo-second-order equations fit well with the experimental data. The nanohybrids exhibit selective adsorption for Cu(II) from multicomponent solutions. These adsorbents show high durability over multiple cycles with desorption efficiency > 93% over six cycles using acidified thiourea. Ultimately, QSAR tools (quantitative structure-activity relationships) were employed to examine the relationship between essential metal properties and adsorbent sensitivities. Moreover, the adsorption process was described quantitatively, using a novel three-dimensional (3D) nonlinear mathematical model.

## 1. Introduction

Water pollution is among the most severe problems facing humanity today, and it has drawn the attention of all scientists. The release of industrial effluent into the aquatic environment is a serious global concern due to the possibility of heavy metal contamination in water reserves [1,2,3]. The mining, electroplating, automobiles, metal processing, textile, and battery manufacturing sectors all contribute significantly to heavy metal pollution. Heavy-metal-containing effluent is routinely released into water bodies as a result of industrial activity, causing a slew of environmental issues [4,5,6,7]. Copper is among the most precious and widely utilized metals in the industry [3,8]. Copper in industrial effluent is particularly dangerous. When ingested in excess, copper accumulation in the liver causes gastro-intestinal problems. Copper poisoning occurs when an excessive amount of copper is consumed, producing nausea, liver and kidney failure, vomiting, and abdominal discomfort [9,10]. The World Health Organization states that the Cu(II) permitted maximum in surface water is 3 mg L^−1^ [9]. Thus, different technologies are frequently applied for removing heavy metals from water, including (i) precipitation, (ii) solvent extraction, (iii) impregnated resins, (iv) ultrafiltration, and (v) adsorption and ion exchange [2,9,11,12]. However, practically all of these techniques are often costly, require multiple stages, are environmentally unfriendly, produce organic wastes, and are ineffective, especially at low metal concentrations [13,14]. Thus, adsorption methods are usually thought to be more effective for treating diluted effluents, due to fast kinetics, reusability, environmental friendliness, selectivity, and high efficiency [1,15,16].

More recently, adsorbent materials such as modified natural materials [1,11], low-cost biopolymers [2,6,17,18], metal–organic-frameworks-based materials [16,19,20], carbonaceous materials (e.g., biochar and activated carbon derived from biomass wastes) [14,21,22], synthetic polymers [23,24], and nanomaterials [18,25] have been used in the field of Cu(II) removal. Biosorption has been proposed as an environmentally acceptable green technique for the elimination of different contaminants [2,26,27,28]. Sustainable biomass with numerous functional groups (–NH_2_ and –OH), renewable resources, and good hydrophilicity are promising and economically feasible alternatives to synthetic polymer adsorbents [9,29,30]. Modified biopolymers based on polysaccharides are excellent examples of these adsorbent types [29]. Chitosan (CS, as an amino-polysaccharide) is a powerful biosorbent for eliminating heavy metals because it possesses a distinct set of characteristics (for example, nontoxicity, biocompatibility, biodegradability, and bioactivity) and is nature’s most abundant and least expensive biopolymer [2,26,30]. To promote adsorption capacity, selectivity, and mass transfer: multiphase nanohybrids were designed as a development of functionalized organic–inorganic nanohybrids [3,24,31]. CS was selected and adapted due to its chemical compatibility with the Fe_3_O_4_ nanoparticles’ fabrication [32,33].

The synergism and mixing between two or more individual components were proposed to design and create new multiphase nanomaterials with improved characteristics, such as multifunctionalization, reaction velocity, stability, and the cost-effectiveness [25,29,30]. This is the driving force behind the development of multifunctionalized chitosan-based hybrid magnetic materials. Magnetic nanohybrids using Fe_3_O_4_ nanoparticles are an innovative type of composite [24,34]. Magnetic-adsorption technologies have been widely employed for contaminants’ removal because it significantly enhances the specific surface area (which reduces restrictions imposed by intraparticle diffusion), and it is rapid, simple, sensitive, and extraordinarily effective. Furthermore, after equilibration is completed, depleted adsorbents can be recovered by utilizing an external magnetic field [31,34,35,36,37]. Until now, no published work has been reported containing the extensive and in-depth description required for mechanistic investigations of the synthesis stages and post-chemical modification, adsorption, and desorption mechanisms for copper with such magnetic multifunctionalized chitosan nanohybrids, which pose different active sites that have not been tested before. Herein, we describe the synthesis of four multifunctionalized chitosan adsorbents based on the functionalization of polymer support (by in situ Fe_3_O_4_ nanoparticles co-precipitation covered with a thin chitosan layer) with different reactive groups via amine polydentate (diethylenetriamine) and amino acids (e.g., alanine/cysteine/serine) grafting. These adsorbents were extensively characterized to justify the synthesis routes (including the iron oxide and organic synthesis mechanism involved in the chemical modification). These functionalization pathways brought a good opportunity for comparing the impact of grafted groups on the physicochemical properties of the materials (including CHNS/O (elemental analysis), HR-TEM (high-resolution transition microscope), XRD (X-ray diffraction), textural properties (using the Brunauer–Emmett–Teller (BET technique) and Barrett–Joyner–Halenda (BJH method)), pH_ZPC_ titration (pH zero-point charge), FTIR (Fourier-transform infrared spectroscopy), TGA/DTA (Thermogravimetric analysis/Differential Thermal Analysis), VSM (vibrating sample magnetometer), EDX (Energy dispersive X-ray Spectroscopy), and XPS (X-ray photoelectron spectroscopy) techniques. The batch experiments were used to investigate the structure activity relationship and compare adsorption performance, selectivity test, and the adsorption–desorption cycle was used to investigate the regeneration and reusability of these nanocomposites. Furthermore, the experimental results were fitted with kinetics and isotherm models (with conventional modeling equations). The adsorption and desorption mechanisms were analyzed and studied using spectroscopic techniques (particularly, FTIR and XPS). Moreover, an effective mathematical method for determining adsorption capacity at varying initial concentrations and pHs was developed using a special modeling methodology for quantitative nonlinear description in three dimensions.

## 2. Materials and Methods

### 2.1. Materials

Chitosan (CS, CAS: 9012-76-4) was purchased from Acros Organics. Diethylenetriamine (99%), 1,4-dioxane (>99%), epichlorohydrin (99%), cysteine (≥98.5%), FeCl_2_.4H_2_O (>99%), alanine (≥98%), FeCl_3_ (>99%), Arsenazo III, and serine (≥99%) were provided from Sigma-Aldrich (Saint-Louis, MS, USA). Every reagent was used as received. Standard atomic absorption solutions (Cu, Co, Ni, Cd, and Zn, 1000 mg/L^−1^ nitrate form in nitric acid) were from Scharlau Chemie (Barcelona, Spain). Samples (5 mL) were centrifuged to separate them. The supernatants were taken, and the remaining Cu(II) concentration was analyzed. Total Zn, Cd, Ni, Co, and Cu concentrations in mixed complex solution were measured using AAS (flame atomic absorption spectrophotometer) (GBC Avanta-EGf 3000, Scientific equipment Ltd., Melbourne, Australia).

### 2.2. Preparation of Adsorbents

**Scheme 1:**
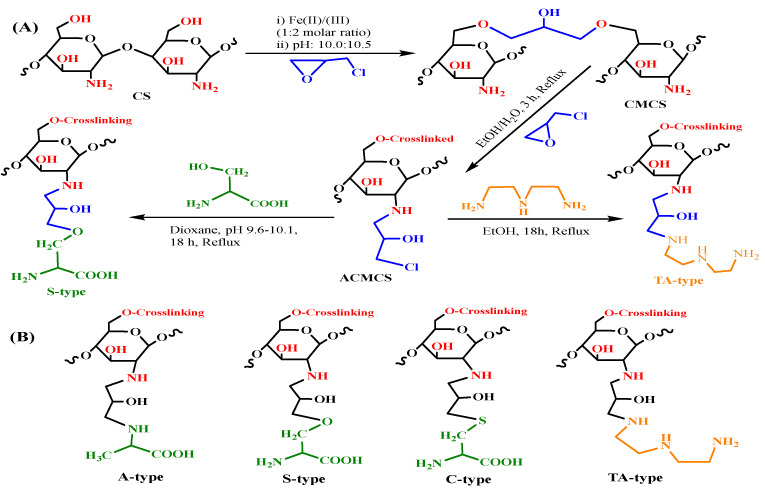
S-type and TA-type nanohybrids synthesis (**A**) and structure of several adsorbent types (**B**).

The adsorbents employed in this study were synthesized according to the previous article, as a foundation, and with minor modifications [25,32]. A schematic representation for the fabrication of magnetic nanohybrid adsorbents is represented as a general method in Scheme 1.

#### 2.2.1. Preparation of Activated Crosslinked Chitosan−Magnetite Nanohybrid

A precipitation method combined with hydrothermal treatment was applied to produce nanohybrid magnetic chitosan particles through a one-step process, which involved in situ co-precipitation of ferrous and ferric ions mixture simultaneously with the dissolved chitosan particles under alkaline conditions [35]. Chitosan solution was firstly prepared by dissolving 5 g of chitosan powder in 600 mL of acetic acid solution (5% *w*/*w*). Then 6.22 g of FeCl_2_ and 9.60 g of FeCl_3_ (with a molar ratio of 1:2, respectively) were added and homogeneously dispersed within the chitosan solution. The resulting solution mixture was subjected to chemical precipitation by the dropwise addition of NaOH (2 M), under steady stirring, at 40–45 °C, and the pH was controlled to 10.0–10.5. The suspension was kept under heating and constant stirring for 1 h at 90 °C before the nanohybrid particles were separated by decantation and magnetic attraction. Secondly, an alkaline solution of 0.01 M epichlorohydrin with pH 10.0 was added to the freshly obtained nanohybrid particles with a mass ratio of 1:1, and the reaction mixture was maintained under heating and continued stirring for 2 h at 50 °C. After that, the resulting material was collected by magnetic separation and washed extensively with ethanol and distilled water to remove any unreacted epichlorohydrin. Finally, the previously obtained crosslinked magnetic chitosan nanohybrid particles were suspended in 200 mL of ethanol/water mixture (1:1 *v*/*v*), followed by the addition of 10 mL of epichlorohydrin, and the mixture was heated and refluxed for 3 h at 70 °C. Thereafter, the activated nanohybrid particles were obtained, filtered, and washed extensively with ethanol and distilled water to remove any residual reagent [32,38].

#### 2.2.2. Nanohybrid Functionalization

The sequential stages for the functionalization of the activated crosslinked magnetic chitosan nanohybrid with diethylenetriamine, as well as amino-acid functionalities, were presented in Scheme 1A. Briefly, the diethylenetriamine moiety was grafted as follows: the epichlorohydrin-activated nanohybrid (2.5 g) was suspended in 50 mL of ethanol, and 25 mL of diethylenetriamine was added. Then the reaction mixture was maintained under reflux for the next 18 h, at 75–80 °C [32,39].

On the other hand, alanine, cysteine, and serine moieties were grafted according to the following procedure: The epichlorohydrin-activated nanohybrid particles (2.5) were suspended in 150 mL of dioxin, and then 6.0 g of alanine/serine/cysteine was added and the pH of the mixture was adjusted to 9.5–10 by using 1 M NaOH solution. After that, the mixture was kept under reflux for the next 18 h at 95 °C [25]. After the reaction, all end-products were collected by magnetic separation and extensively washed with ethanol and double-distilled water. Finally, the obtained adsorbent materials were freeze-dried for at least 24 h.

### 2.3. Characterization Techniques

The obtained superparamagnetic multifunctional chitosan adsorbents were characterized by HR-TEM, CHNS/O, XRD, BET, pH_ZPC_ titration, FTIR, TGA/DTA, VSM, EDX, and XPS techniques. More detailed information for the different characterizations is offered in Supplementary Materials Section S1. To examine the material’s chemical stability, a simple technique for testing and evaluating the chemical resistivity and durability of the nanohybrids was achieved by leaching under acidic and alkaline media [25]. So, various pHs (1.0–10.0) were used to submerge and then leach these materials (mass 10.0 mg/10 mL solution, at 25 °C for 48 h). After that, the treated samples were filtered and thoroughly washed (using deionized water), and both treated and untreated samples were dried for 8 h at 75 °C. The mass loss efficiency was calculated as follows: (∆wt% = ((m_0_ − m_eq_) × 100)/m_0_), where m_0_ is the untreated dry mass, and m_eq_ is the treated dry mass. After several adsorption/desorption cycles, the nanohybrid materials were further examined for functional stability, using FTIR spectroscopy.

### 2.4. Adsorption Experiments

Batch adsorption tests have been performed at a fixed adsorbent dose (0.5 g.L^−1^) to investigate the pH influence (the pH_0_ ranging from 2.0 to 7.0), agitation time (time was varied from 0 to 360 min at pH_0_ 5.0), sorption isotherms (at initial Cu(II) concentration from *C*_0_: 0.128 to 1.243 mmol Cu.L^−1^ at temperatures ranging from 298 to 328 ± 2 K, and pH_0_ 5.0), and adsorbent reusability at 298 ± 1 K and 150 rpm. The adsorption capacity (q_eq_, in mmol Cu.g^−1^) and the distribution coefficient (D, in L.g^−1^) were evaluated using the following equations (Equations (1) and (2), respectively):

(1) $$q_{\mathrm{eq}}=\frac{(C_{o}-C_{\mathrm{eq}})\times \;V}{m}$$

(2) $$D=\frac{q_{\mathrm{eq}}}{C_{\mathrm{eq}}}=\frac{\left(C_{o}-C_{\mathrm{eq}}\right)}{C_{o}}\times \frac{V}{m}$$

where C_0_ and C_eq_ (in mmol Cu.L^−1^) refer to the initial and remaining Cu(II) concentration after equilibrium, m (in g) points to is the adsorbent amount, and V (in L) corresponds to the volume.

Investigating the selectivity concerns in complicated solutions is required for assessing the potential of novel materials. A standardized study was carried out using the most common ions found in industrial wastewater, e.g., those from battery plants (such as Cu, Co, Zn, and Cd). For this test, an equimolar mixed solution was prepared (using standard atomic nitrate solutions). The adsorption test was conducted at pH_0_ 5.0, for 4 h, at room temperature (298 ± 1 K) and 150 rpm, with an adsorbent dose of 0.5 g.L^−1^. The supernatant concentrations were determined by AAS after centrifugation and separation. The adsorption characteristics could also be modulated by utilizing some chosen models (as shown in Supplementary Table S1) to match the adsorption kinetics and isotherms.

The adsorbent reusability was investigated after six adsorption–desorption cycles utilizing acidified thiourea (0.25 mol.L^−1^ at pH: 2) as eluent and regeneration agent (SD: 1 g.L^−1^, 150 rpm and 298 ± 1 K for 60 min).

The desorption efficiency (D_E_,) and the regeneration efficiency (RE) were described using the equations below (Equations (3) and (4)):

(3) $$D_{E}=\frac{C_{D}{\;\times \;V}_{L}\;\times \;100}{m_{d\;}{\times \;q}_{d}}$$

(4) $$\mathrm{RE}=\frac{q_{d}\;\times \;100}{q_{e}}$$

where q_d_ (in mmol Cu.g^−1^) points to the first adsorption capacity, C_D_ (in mmol Cu.L^−1^) refers to eluate Cu(II) concentration, V_L_ relates to the eluent volume, and m_d_ (in g) represents the adsorbent weight used in desorption tests. All data are means of duplicates with a standard deviation of ±4–6%.

## 3. Results and Discussion

### 3.1. Fabrication Mechanism

Scheme 1A depicts the synthesis steps of multifunctionalized magnetic chitosan nanohybrid, whereas Scheme 1B depicts the chemical structure of the various derivatives. The nanohybrid was formed via heterogeneous nucleation through an in situ hydrothermal co-precipitation of Fe^2+^ and Fe^3+^ in chitosan solution at a pH of ~10.5 with NaOH solution. Firstly, Fe_3_O_4_ nanoparticles were utilized as a nucleation site for chitosan thin-layer formation [35]. The fundamental general process for producing mixed iron oxide (i.e., magnetite (Fe_3_O_4_)) growth of nanoparticles begins after NaOH is introduced to iron solutions, as shown in the following procedures in Equations (5)–(10) [25,40].

Deprotonation step:

(5) $${\mathrm{Fe}}^{3+}+{\mathrm{xH}}_{2}O\;\to 2\mathrm{Fe}{\left(\mathrm{OH}\right)}_{x}^{3-x}+{\mathrm{xH}}^{+}$$

(6) $${\mathrm{Fe}}^{2+}+{\mathrm{yH}}_{2}O\;\to \mathrm{Fe}{\left(\mathrm{OH}\right)}_{y}^{2-x}+{\mathrm{yH}}^{+}$$

Ferrihydrite intermediate production:

(7) $$2\mathrm{Fe}{\left(\mathrm{OH}\right)}_{2}^{+}+\;\mathrm{Fe}{\left(\mathrm{OH}\right)}^{+}+3{\mathrm{OH}}^{-}\to {\left({\mathrm{Fe}}^{3+}\right)}_{2}\left({\mathrm{Fe}}^{2+}\right){\left({\mathrm{OH}}^{-}\right)}_{8}$$

Oxidation and dehydration step:

(8) $$2\mathrm{Fe}{\left(\mathrm{OH}\right)}_{x}^{3-x}+\mathrm{Fe}{\left(\mathrm{OH}\right)}_{y}^{2-y}\;\to {\mathrm{Fe}}_{3}O_{4}+\frac{2x+y}{2}H_{2}O$$

When ferrihydrite dehydrates, magnetite forms:

(9) $${\left({\mathrm{Fe}}^{3+}\right)}_{2}\left({\mathrm{Fe}}^{2+}\right){\left({\mathrm{OH}}^{-}\right)}_{8}\to {\mathrm{Fe}}_{3}O_{4}+4H_{2}O$$

Overall reaction:

(10) $$2{\mathrm{FeCl}}_{3}+{\mathrm{FeCl}}_{2}+8\mathrm{NaOH}\;\to {\mathrm{Fe}}_{3}O_{4}+8\mathrm{NaCl}\;+4H_{2}O$$

This reaction occurs in the presence of N_2_(g) to prevent Fe^2+^ from converting to Fe^3+^; otherwise, different ferric hydroxide forms, such as maghemite, hematite, and goethite, can be formed by oxidizing magnetite [40].

Chitosan particles were crosslinked using epichlorohydrin to prevent them from dissolving under acidic environments and from losing adsorption ability when amine groups participated in the case of aldehyde linkage [41]. Indeed, the unfunctional crosslinking agent was employed to give covalent connections with the C_6_–OH in chitosan moiety, resulting from the epoxide ring breakage and the release of a chlorine atom [25].

**Scheme 2:**
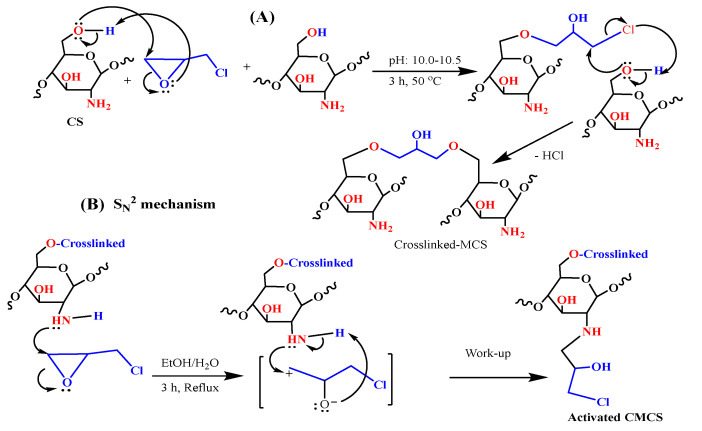
Revised process for the epichlorohydrin crosslinking chitosan formation (**A**) and epoxy ring opening with crosslinked MCS (**B**).

The plausible mechanism for the formation of the crosslinked chitosan (as depicted in Scheme 2A) involves the nucleophilic ring opening of the epoxy ring of the epichlorohydrin under basic conditions by primary OH of the less steric hindrance (C_6_–OH). The second step involves the nucleophilic displacement of the chloride by the nucleophilic primary hydroxyl group of the second chitosan molecule from another chain, followed by the elimination of the HCl under basic conditions after forming the crosslinked chitosan. After that, the activation phase provides active spots on the surface of chitosan, allowing for chemical modification with amine and amino acid moieties.

Scheme 2B shows the S_N_^2^ pathway (S_N_^2^: substitution nucleophilic bimolecular), which might have a role in the nucleophilic assaults of the epoxy ring on nitrogen (CS–NH_2_). A carbonium ion is formed when an N-atom attacks the external carbon (which is less sterically inhibited).

**Scheme 3:**
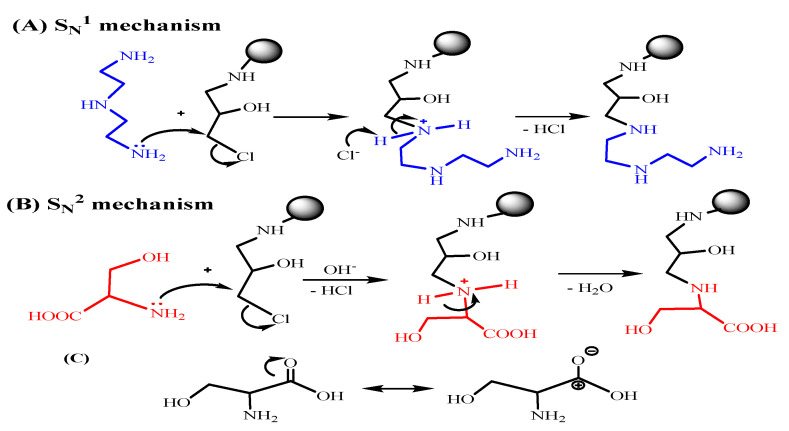
The revised procedure for amine immobilization via S_N_^1^ mechanism (**A**) and for serine (as reprehensive for amino acids) grafting via S_N_^2^ mechanism (**B**,**C**).

Scheme 3 shows two pathways (A (S_N_^1^) or B (S_N_^2^)) which could be involved in the nucleophilic displacement of –Cl with electron-rich terminal hetero atoms (e.g., nitrogen (present in –NH_2_), oxygen (as –OH in serine), and/or sulfur (as –SH in cysteine). For diethylenetriamine grafting, the postulated mechanism of the amination (Scheme 3A) is a typical substitution nucleophilic unimolecular (S_N_^1^) reaction that is preferred in protic solvents such as ethanol [39]. Chloride nucleophilic displacement by electron-rich nitrogen of the amine occurs via the formation of carbocation intermediates, and it is maintained and supported by ethanol solvation and hence favors product formation.

Meanwhile, for amino-acid grafting (Scheme 3B), under alkaline conditions and heating, the usual S_N_^2^ reaction takes place between nucleophilic sites available at the terminal –NH_2_ and/or –SH/–OH on alanine/cysteine/serine–amino acid and the C-atom connected to the Cl atom that is considered to become the promoter for the chemical reaction [25,38]. This reaction is enhanced by an alkaline aprotic solvent such as dioxane. For cysteine/serine grafting, the carboxylic group is an electron-withdrawing site that contributes to the reduction of the N-lone atom’s electron pair, trying to make them lesser nucleophilic (Scheme 3C); this may explain why other –SH/–OH groups are favored versus –NH_2_ where possible. Meanwhile, the –NH_2_ group seems to be more active and preferred in alanine, despite the fact that certain amino groups may be implicated in the reaction. The majority of the grafting seems to occur using an alternate substituent via –SH/–OH active sites.

### 3.2. Materials Characterization

#### 3.2.1. Physical Characterization

##### Nano-Structure Characterization—HR-TEM Analysis

**Figure 1:**
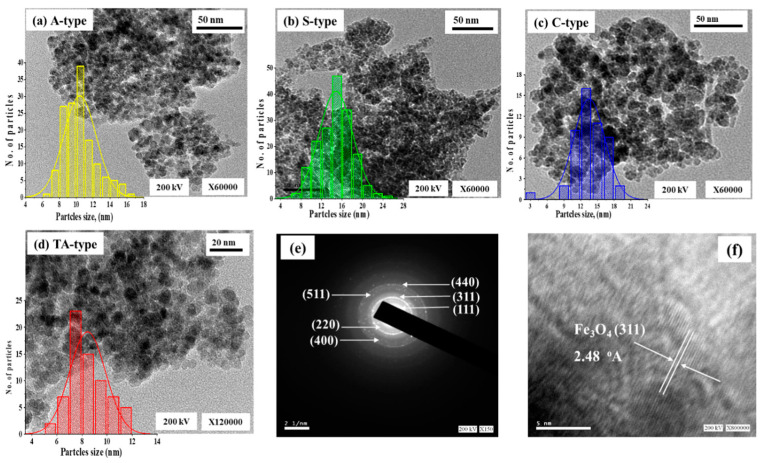
Particle sizes distribution using TEM micrographs and histograms for A-type, S-type, and C-type (**a**–**c**), and for TA-type (**d**), SAED analysis (**e**), and crystal lattice HRTEM picture (**f**).

The TEM micrographs of four adsorbents (A-type, S-type, C-type, and TA-type) were shown in Figure 1a–d. The Fe_3_O_4_ nanoparticles formed as compact spherical spots encircled by bright regions represented the polymeric shells [42] (due to the differences in electron-absorbing capacities of organic and inorganic components [34]); they have a uniform spherical morphology, are finely shaped, and have a mono-dispersed appearance. As a result of dipole/dipole magnetic interactions, the Fe_3_O_4_ particles pose an affinity to coalesce and agglomerate [41,43]. This exhibits the incorporation of Fe_3_O_4_ NPs into the polymer matrix, which was successfully prepared. The clumping validates the great magnetism, just like it was evaluated via magnetism tests. TEM images were used to determine and identify the exact the Fe_3_O_4_ nanoparticle size. The mean crystalline size for A-type, S-type, C-type, and TA-type was estimated to be 10.43 ± 1.9, 14.79 ± 3.2, 14.01 ± 3.7, and 8.45 ± 1.4 nm, utilizing histograms image software to count particles with a limited size distribution (Figure 1a–d), in line with XRD findings. In general, the diameter of Fe_3_O_4_ particles is smaller than 20 nm, suggesting that the adsorbents are nanocomposites.

The HR-TEM picture (Figure 1e,f) for TA-type, according to the SAED (selected area electron diffraction) pattern, exhibits that particles majorities visible and found in the bright field image with an ordered spherical shape (Figure 1e). This pattern has an inverted spinel structure (magnetite/hematite), which supports the two phases deduced from the XRD [39]. It is clear by precisely examining the SAED pattern that the spinel structure peak (311) related to the first intense magnetite broad ring of magnetite (or hematite) displays an outstanding efficient epitaxial formation of Fe_3_O_4_ single crystals [39,44]. Furthermore, the (511) and (220) planes of the Fe_3_O_4_ spinel structure were discernible. According to the HRTEM image, the nanoparticle ensembles are composed of a number of single crystals with polycrystalline particles (Figure 1e). Interestingly, the inter-planar distance (311) of the Fe_3_O_4_ planes is consistent with the crystalline fringe spacing of 0.248 nm (~2.48 Å) [24,44].

##### Crystalline Structure—XRD Analysis

**Figure 2:**
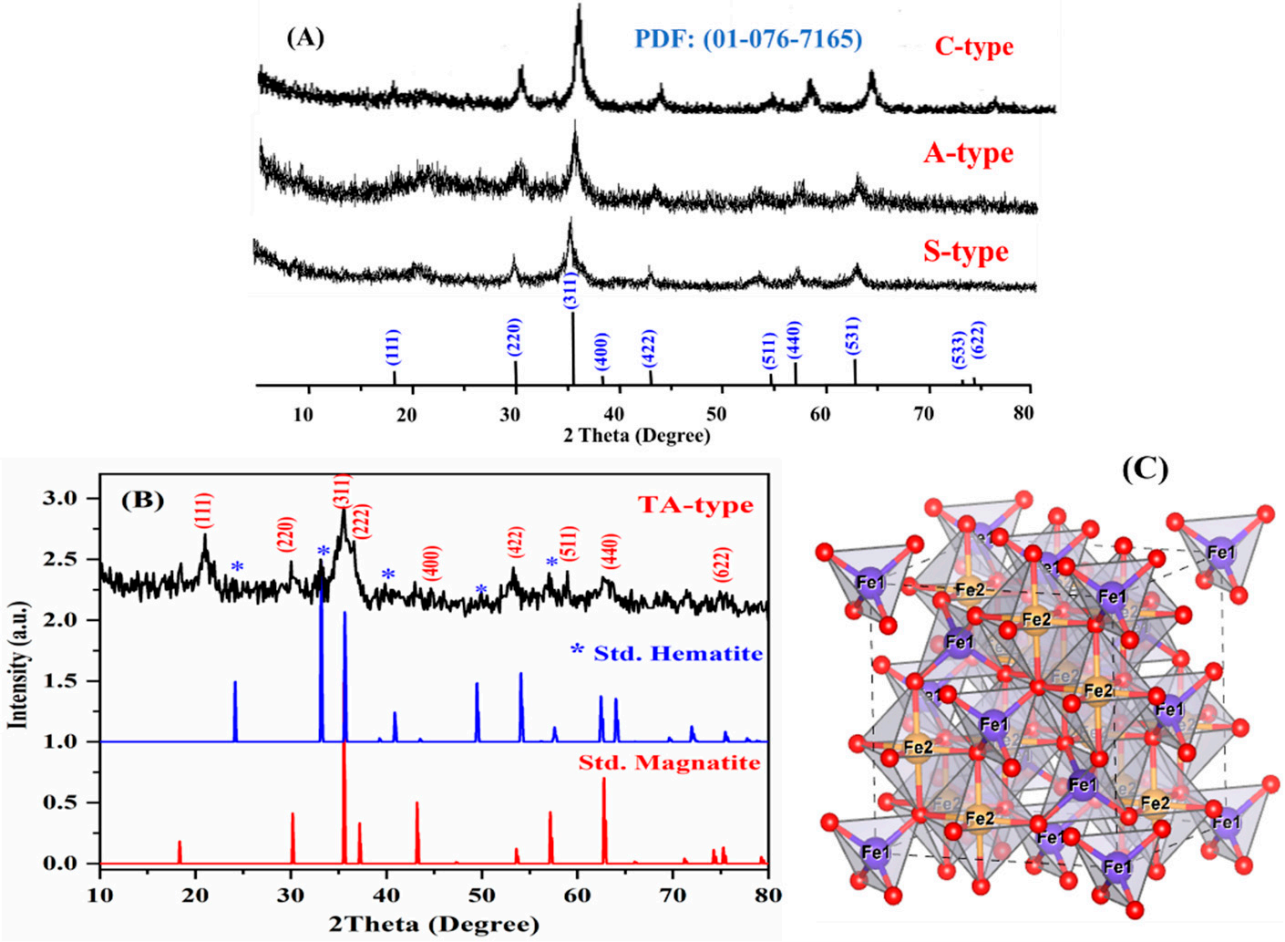
XRD analysis of the four different nanohybrids (**A**,**B**). The bar-type spectrum depicts the standard magnetite and hematite pattern (**B**). The spinel structure’s unit cell crystal structures (**C**).

The XRD spectra of the as-prepared nanohybrids are shown in Figure 2: magnetite (Fe_3_O_4_) has a crystalline cubic spinel shape [43]. Based on the JCPDS (PDF No. 65-3107) database file, all nanohybrids contain eight typical diffraction peaks for Fe_3_O_4_ at 2θ around 19°, 30°, 35°, 43°, 54°, 57°, 62°, and 74° [39,45]. Furthermore, the possibility of hematite (Fe_2_O_3_) coexisting with ferromagnetic properties cannot be left out., according to the findings of the XPS study, which is discussed later. The Fe2*p* band in the adsorbents (TA-type and C-type) spectrum may be deconvoluted by XPS into three signals (each signal having a unique doublet band) associated to Fe^3+^ in both octahedral (at BEs ~710.27–710.68 eV, ~712.81–713.23 eV, and ~732.42–732.39 eV for octahedral geometry) and tetrahedral (at BEs: 716.45–716.96 eV and ~727.12–727.69 eV), and Fe^2+^ (at BEs ~723.80–724.31 eV for tetrahedral geometry). The relevant satellite bands have a low resolution. Moreover, an Fe2*p*_3/2_ signal (Fe^3+^- octahedral form) at roughly 711.0–711.6 eV might be assigned to the most prevalent kinds of iron oxides, such as hematite (Fe_2_O_3_) or maghemite (g-Fe_2_O_3_) [46].

The SAED pattern indicated the nanohybrids’ polycrystalline structure, which is characterized by the presence of sharp and continuous loops (Figure 1e). Furthermore, XRD spectra were used to evaluate the diameters of the crystalline Fe_3_O_4_ nanoparticles by using the Debye–Scherrer Equation: (*D* = kλ/βCosθ) [36,47], where *D* is the mean particle diameter (in nm), k is the shape factor (0.9), λ is X-ray radiation wavelength (~1.5418 Å), θ is the diffraction angle, and β is the FWHM (full width at half maximum) of the selected X-ray diffraction peaks. The size was estimated to be around 9.43, 10.83, 9.78, and 9.01 nm for A-type, S-type, C-type, and TA-type, respectively, for the most intense (311 index) peak (validated by SAED picture) [38].

From the aforementioned findings, Figure 2C depicts the structural Fe_3_O_4_ characteristics. Magnetite is composed of Fe^2+^Fe_2_^3+^O_4_ (generally so-called mixed oxide “Fe_3_O_4_”), where Fe1 and Fe2 are associated with Fe^2+^ and Fe^3+^, respectively, showing cation positions inside the octahedron and tetrahedron, respectively, and O denotes the oxygen anion position. The unit cell of spinel magnetite is a cube composed of 32 O^2−^ anions that are formed by 8 Fe^2+^Fe_2_^3+^O_4_ molecules [39]. The packed face-centered cubic of Fe_3_O_4_ (FCC) is composed of oxygen anions that comprise 64 Fe1 (tetrahedral) and 32 Fe2 (octahedral) vacant spaces that are partially occupied by Fe^3+^ and Fe^2+^ cations [48].

##### Textural Properties—BET Surface Analysis

The textural features of the as-prepared nanohybrids were assessed using the BET method: N_2_(g) sorption (i.e., via the adsorption–desorption) and isotherms. Nitrogen isotherms of nanohybrid derivatives mirror Type-IV isotherms associated with mesoporous adsorbents (Supplementary Figure S1) [38,49]. The specific surface areas calculated using the BET method (S_BET_ in m^2^.g^−1^) for all nanohybrid derivatives were found and follow the following sequence: TA-type (75.27) > S-type (61.05) > A-type (~60.38) > C-type (~42.56). Moreover, the textural qualities of all nanohybrids are essentially similar: likely to be mesoporous in structure. The typical pore diameters are ~27.5, 26.9, 22.5, and 3.9 nm for TA-type, S-type, A-type, and C-type. However, the significant whole pore volume (WPV in cm^3^.g^−1^) was recorded: TA-type (0.57) > S-type (0.41) > A-type (0.36) > C-type (0.12).

##### Thermogravimetric Analysis (TGA)

The thermogravimetric properties of S-type are depicted in Figure 2A. Significant variations could be found in terms of weight loss and the numbers of degradation stages, as well as in the DTG diagrams (Supplementary Figure S2a,b). The deterioration profile is distinguished by four transitions: (a) The first phase (until 202–206 °C) is concerned with the liberation of absorbed water molecules at the adsorbent surface [47], and the consequent structural changes resulted in a 12% weight decrease. (b) The second phase (within 206–350 °C) results in a weight loss of 25–27% (cumulative wt. loss: 37–39%) [17], which is linked to the decomposition and destruction of amine moieties in chitosan (itself) and its amine and amino acid derivatives [35]. (c) The third phase (from 350 and 439 °C) resulted in a weight loss of around 4–7% (max. wt. loss: 41–44%) and includes depolymerization and pyranose rings disintegration [47]. (d) The final phase (up to 650 °C) corresponds to the char entire disintegration, which accounts for about 56.2%. This fraction corresponds to the magnetic core amount in the composites. The tracker of magnetite with char remaining at higher temperatures may throw this judgement off [35].

Furthermore, the DTG graph (Supplementary Figure S2b) indicates three significant valleys, namely at ~234.0 °C (strong and abrupt), at ~345.1 °C, and at ~415.2 °C, as well as a mild valley at 502.5 °C, related to the variations in the TGA profile slope and the release of adsorbed and structured water (where the samples were freeze-dried gave the strongest valleys), the breakdown of amine and amino acid moieties in adsorbent (i.e., chitosan and the derivatives), the depolymerization and breakdown in pyranose ring accompanying char formation, and, eventually, the char’s thermal deterioration (final portion of deterioration) [35,47].

##### Magnetic Properties—VSM Analysis

The magnetic profiles of MCS and their derivatives were evaluated and analyzed using VSM (Supplementary Figure S2c). The hysteresis curves of all nanohybrids materials exhibit extremely small remanence magnetization (*M*_r_), coercivity field (*H*_c_), and no hysteresis loop (Supplementary Table S2: the basic physicochemical features of these nanohybrid materials; that is, the magnetization of the residue was almost zero, indicating superparamagnetic behavior. The magnetic saturation (*M*s) is 42.97 emu.g^−1^ for MCS; nevertheless, after chemical modification via amine and amino acids immobilization, the *M*s values (in emu.g^−1^) were reduced and arranged as follows: r-MCS (42.97) > TA-type (31.13) > A-type (26.94) > S-type (23.21) > C-type (22.84). Because *M*s values are connected to the amount of Fe_3_O_4_, the organic coating thickness increases with further chemical functionalization, resulting in a reduction of the iron oxide quantity and, hence, a decrease in *M*s values [25,39]. Meanwhile, these *M*s values are sufficient for simple and efficient magnetic separation from aqueous solutions [38,39].

#### 3.2.2. Chemical Characterization

##### XPS Spectroscopy

**Figure 3:**
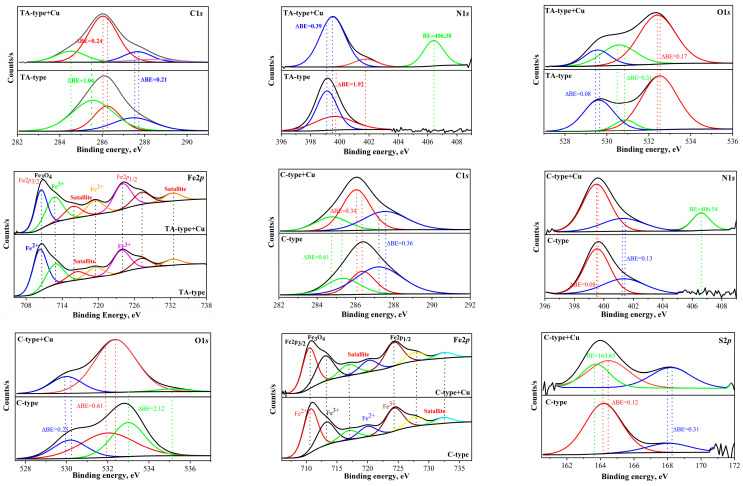
HR-XPS of TA-type and C-type for (C1*s*, N1*s*, O1*s*, Fe2*p*, and S2*p* spectra) before and after Cu(II) adsorption.

The XPS survey signalizes the elemental composition of all nanocomposite derivatives (Supplementary Figure S3): C1*s,* N1*s*, O1*s*, Fe2*p*, and S2*p* signals at the binding energy (BE) of 286.97–288.29 eV, 400.17–401.37 eV, 532.74–533.88 eV, 711.96–713.41 eV, and 165.5 eV, respectively. Furthermore, two peaks at 710.3–711.4 eV (Fe2*p_3/2_*) and 725–726 eV (Fe2*p*_1/2_) distinguished the magnetite. The core levels’ profiles for essential components are depicted in Figure 3 (and Supplementary Table S3). The deconvoluted spectra were determined by the assignments and average atomic fractions [50]. The C1s curve figures out the peaks at 285.6 (corresponding to C–H, C–C, C_advent_), 286.3 (related to C–OH, C–N, C–O–C, C–S), and 287.5–288.8 (attributed to C=O (Amide), and –COOH). The N1s spectrum represents the N– containing functional groups since the related peaks to (N–H, C–N (amide) and (–NH_2_, >NH (amine)) appeared at BE of ~399.3 and ~400.3 eV, respectively. A slight shift for amino acid derivatives was observed that could be due to the carboxylate environment. The O1s curve depicts the belonging peaks at BEs: 529.61 eV (corresponding to Lattice O (Fe–O, Fe_3_O_4_)), 530.87 eV (refers to C=O, –O–C=O), and 532.51 eV (related to C–OH, O–H in H_2_O, and C–O–C). The S2*p* graph depicts the S–containing functional groups at BEs: 164.17 eV (C-S (2p_3/2_)) and at 168.05 eV C-S-C (S2p_1/2_).

The primary peaks of Fe^3+^ 2*p*_3/2_ and Fe^3+^ 2*p*_1/2_ that are considered to be distinct Fe_3_O_4_ are located at ~710.27–711.67 and ~723.80–724.60 eV, respectively, in the Fe2*p* spectrum (Figure 3) [37,51]. After fitting the Fe2*p* dual peaks, we noted that Fe^2+^2*p*_3/2_ peaks emerge at ~712.16–713.02 eV and Fe^3+^ 2*p*_1/2_ peaks appear at ~719.43–720.21 eV, confirming the existence of Fe^2+^ and Fe^3+^, respectively, corresponding to Fe_3_O_4_. The Fe^2+^ 2*p*_3/2_ peaks appear at ~712.16–713.02 eV, while the Fe^3+^ 2*p*_1/2_ peaks arise at ~719.43–720.21 eV, proving the coexistence of Fe^2+^ and Fe^3+^, respectively, which corresponds to Fe_3_O_4_ [37], whereas the Fe^2+^ 2p_1/2_ satellite peak occurs at ~723.81–725.01 eV [37,52].

##### Element Analysis-CHNS/O

The effective chemical modification of chitosan backbone was confirmed using CHNS. Supplementary Table S2 summarizes and compares the elemental analysis of the various samples. The organic fraction (associated to C and N, wt. %) of raw chitosan (rCS) and magnetic chitosan (MCS) was examined and found to be substantially lower (52.7–53.9%) for rCS because of the magnetite content that represents ~53.5% of overall mass. After crosslinking process, the N-content (in mmol%) of crosslinked magnetic chitosan (CMCS) reduced from 2.56 to 2.17, owing to increased epichlorohydrin binding, which is consistent with the small decreases in the C and H contents. In the meantime, after activation via terminal –Cl atom insertion, the activated matrix (ACMCS) molar mass increased steadily; thus, the wt.% of C, H, and N was diluted and reduced [25]. Subsequently, immobilization of multifunctional moieties: the rise in C, H, N, and S content (in wt/wt) indicated the successful functionalization and grafting onto ACMCS. More particularly, the N content virtually increased from 1.41 mmol N.g^−1^ to 2.69, 2.58, 2.35, and 3.72 mmol N.g^−1^ for A-type, S-type, C-type, and TA-type, respectively [25,39]. Assuming C-type, the S content (in wt,%) of 3.01% supports effective cysteine grafting [38].

To estimate magnetite proportion of nanohybrid materials, the thermal degradation characteristic of the S-type was used (see Section Thermogravimetric Analysis–TGA). The S-type was a stand-in for distinct derivatives, since all nanohybrids have the same matrix and the only change is in the grafted moieties. The magnetic core portion accounted for 48–52 ±0.5% of total weight. This fact is critical when considering the adsorption performance, as the inorganic fraction contributes half the total weight of the adsorbents and has a lower affinity for Cu(II) ions than the organic component of chitosan derivatives.

##### FTIR Spectroscopy

**Figure 4:**
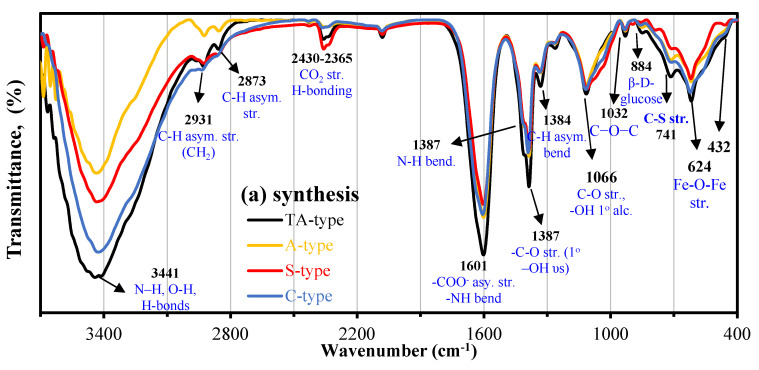

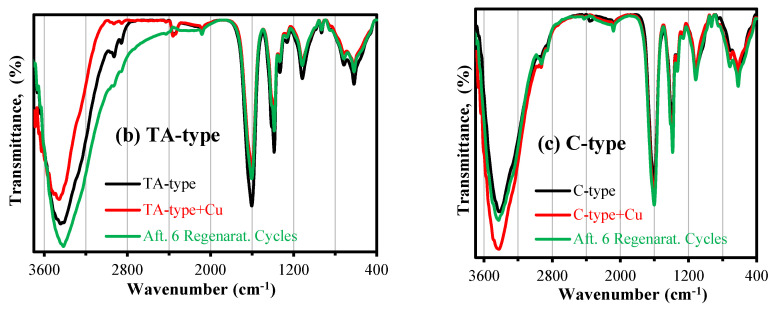
FTIR spectra (**a**) for the synthesized nanohybrids; (**b**) TA-type; and (**c**) C-type, pristine, after Cu(II) adsorption, and after 6th regeneration cycle.

Synthesis stage: to characterize the functional groups of the nanohybrid adsorbents, infrared spectroscopy was utilized and is presented in Figure 4a. The spectra were essentially all fairly similar; the primary distinctions were in the relative strength. The band at 616–627 cm^−1^ corresponds to the υFe–O stretching vibration in Fe_3_O_4_ [25,36]. The peaks at ~533–558 cm^−1^ and ~456–480 cm^−1^ result from the split of the υ_1_(Fe_tetra_–O band) and υ_2_(Fe_octa_–O band) which is the blue-shift of bulk Fe_3_O_4_, respectively [25,31]. Generally, the strong and broad absorption peaks at ~3468–3400 cm^−1^ are relevant to the stretching vibration for –NH_2_/>NH and –OH stretching vibration (and their overlapping) and hydrogen bonds in polysaccharides [31]. The peaks at 2923–2936 cm^−1^, 2852–2869 cm^−1^, and 1387–1392 cm^−1^ are assigned to C−H symmetric and asymmetric stretching vibration, respectively (in –CH_2_ (methylene groups)), and C–H bending, respectively [26,35,53]. The peak around 2432–2334 cm^−1^ is caused by the CO_2_ vibration. The substantial peak at 1602–1610 cm^−1^ could be ascribed to >NH bending vibration (−NH_2_ and >NH amine bend, in plane deformation), and C=O (Amide I) and/or (COO^−^) carboxylate group symmetric stretching vibration [23,30]. The bands at 1330–1256 cm^−1^ (referring to C–H (in CH_2_) symmetric deformation and stretching vibration of C−N) and the ranges between 1112 and 11161 cm^−1^ and between 936 928 cm^−1^ (corresponding to stretching vibration of secondary –OH and C–O–C (in β–glucosidic bridge) [31], and β-D-glucose unit of carbohydrate ring) [54]. The peaks at 854–798 cm^−1^ and 722–711 cm^−1^ (corresponding to stretching vibration of secondary –OH and C–O–C bridge [54],) are caused by C-H out-of-plane bend bending vibration of C–H and –NH twist, respectively [26,35,39]. The C–C–N out-of-plane bending mode was assigned to the weak band at 464–480 cm^−1^ [23,55]. The absence of the C–S functional group’s peak at 742 cm^−1^ could be due to the overlapping with other groups [41].

**Scheme 4:**
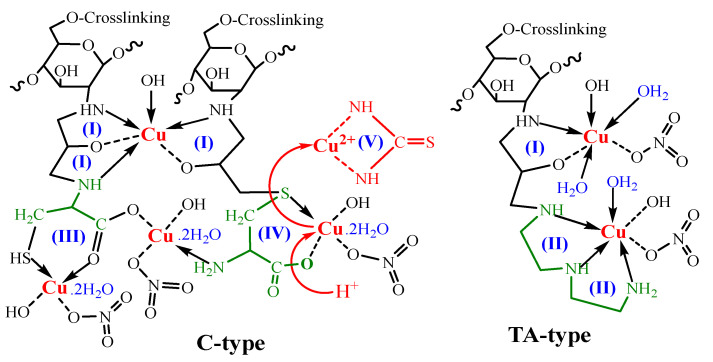
Interaction and desorption mechanism of TA-type and C-type with Cu(II) complexes.

FTIR for characterization of Cu(II) interactions with TA-type and C-type nanohybrids: The FTIR spectra for both TA-type and C-type adsorbents are affected by Cu(II) adsorption (Figure 4b,c and Supplementary Table S4) as follows:

(a) For TA-type: The bands’ intensities tend to strongly decline and red-shift at 3468, 3441, and 3398 cm^−1^ (assigned to −OH and −NH functionality); 2936 and 2857 cm^−1^ (–CH_2_ groups); 1605 cm^−1^ (υsC=O (Amide I), (1°/2°) amine bend and their overlapping); and 850 cm^−1^ (C–H out-of-plane bend), shifting to 3467, 3436, 3391, 2923, 2858, 1602, and 818 cm^−1^, respectively. Meanwhile the blue-shift at 1326–1260 cm^−1^ (υC–N stretching), 1113 cm^−1^ (C–O–C bridge υs), 928 cm^−1^ (υsC–O, 1° OH group, carbohydrate ring, and υsC-N), 710 cm^−1^ (−NH twist), 533 cm^−1^ (υ_1_Fe_tetra_–O), and 464 cm^−1^ (υ_2_Fe_octa_–O) moved to (1330–1261 cm^−1^), 1117 cm^−1^, 932 and 714 cm^−1^, 541 and 472 cm^−1^, respectively.

(b) For C-type: The bands’ intensity alteration and blue-shift at 3436 cm^−1^, (υs (–OH and –NH), 1404–1392 cm^−1^ (υsCH_2_ def., (1°/2°) –OH bend, and COO^−^ salt), 1330-1265 cm^−1^ (υC-N and –OH bending, –C–O str. (primary υs(–OH)), 1113 cm^−1^, 855 cm^−1^, 1113 cm^−1^, 722(−NH twist), 627 (υsFe–O–Fe and C–H bending), and 591 and 480 cm^−1^, (υ_1_Fe_tetra_–O and υ_2_ Fe_octa_–O, respectively) shifted to 3416 cm^−1^, 1400–1387 cm^−1^, 1329–1260 cm^−1^, 1112 cm^−1^, 853 cm^−1^, 714 cm^−1^, 624 cm^−1^, 541 cm^−1^, and 456 cm^−1^. Meanwhile, the red-shift was at 2927 and 2857 cm^−1^ (–CH_2_ groups), 1609 (υsC=O (Amide I), COO^−^ salt, (1°/2°) amine bend and their overlapping), and 932 cm^−1^(υsC–O, 1° OH group, and υsC-N) to 2931 and 2858 cm^−1^, 1610 cm^−1^, and 936 cm^−1^. The reactive groups involved in metal interaction were identified and discussed with other XPS and adsorption results and are presented in Scheme 4 (and Section 3.3.7).

In both adsorbents (TA-type and C-type), numerous bands related to the stretching vibrations of M−OH and O−M−O were seen in the FTIR spectra at the 400–800 cm^−1^ range [23,35]. These changes were caused by Cu(II) binding through complexation with various reactive groups that alters the environment.

FTIR spectra after Cu(II) desorption: Figure 4b,c (and Supplementary Table S4) also display the TA-type and C-type nanohybrids’ spectra after six regeneration cycles (adsorption/desorption of copper). The adsorbents’ FTIR spectra were little impacted. Although the copper desorption was effective (Section 3.3.5), several tracer bands associated with some attached Cu(II) ions remained on each regenerated adsorbent’s FTIR spectrum, whose spectrum between copper loaded and raw adsorbents can be thought of as “intermediary.

##### pH_ZPC_—Drift Titration

Supplementary Figure S2d displays the rCS, MCS, and multifunctionalized nanohybrid titration patterns. The consecutive chemical changes have a significant impact on the acid–base properties [35]. The pH_ZPC_ (is equivalent to ∆pH = 0) for rCS is 8.57 ± 0.02, and it correlates to the p*K*a of 6.3–6.6 for chitosan’s –NH_2_ groups [26]. After the incorporation of the Fe_3_O_4_ core in MCS, the pH_ZPC_ dropped to 7.26 ± 0.03. The triamine/alanine/cysteine/serine immobilized onto MCS nanohybrid hardly influences the pH_ZPC_: from 7.26 ± 0.03 for MCS to 7.88 ± 0.01, 7.66 ± 0.02, 7.78 ± 0.03, and 8.48 ± 0.02 for A-type, S-type, C-type, and TA-type, respectively, consistently with charge density, screening impact, and Fe_3_O_4_ amount [30]. The maximal obvious rise in ∆pH is at pH_0_ 4.0, ascribed to the maximum protonation degree, which declines as the pH_0_ increases.

Surely, after triamine/alanine/serine/cysteine grafting, the pH_ZPC_ increased, most likely as a result of the extra functionalities grafting (e.g., amine and carboxylate moieties). Amino acids contain carboxylic acid groups having p*K*a values ranging from 2.11 to 2.35, whereas their amine groups have p*K*a values ranging from 9.15 to 9.69 [25]. The overall pH_ZPC_ of amino acid nanohybrids were closest to those of the amine group of alanine/cysteine/serine in comparison to the carboxylate group end. After diethylenetriamine, immobilization was proportionate to the pKa values 3.58, 8.86, and 9.65 of the amine active sites [56]. Fraga et al. measured the pH_ZPC_ for DETA grafted on graphene oxide, and it was found to be 8.21 [57], wherein the ∆pH_ZPC_ was 0.29 pH unit (the change achieved 8.48), as a result of the support impact switched from graphene oxide to chitosan.

##### Chemical Analysis—Semi-Quantitative EDX

**Figure 5:**
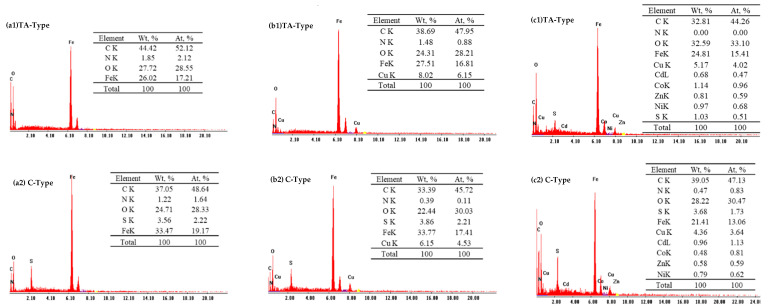
EDX measurements of TA-type and C-type adsorbents before (**a**) and after Cu(II) adsorption from synthetic solution (**b**) and complexed system (**c**).

The EDX graphs of nanohybrid materials (Figure 5 and Supplementary Figure S4) exhibited the appearance of C, N, O, and Fe signals, with an additional S signal for the C-type. The EDX analysis indicates the existence of the characteristic peak for K_α_ Fe (at 6.403 keV) and L_α_ (poor peak) at 0.705 keV, as well as the emergence of a minor peak (having a low sensitivity signal at 0.393 keV for the K_α_ N), thus validating the hybrid formation. The O k_α1_ signal (at 0.525 keV) and C k_α_ signal (at 0.277 keV) were observed. Moreover, the S element in the C-type is identified by S k_α_ and S L_α_ signals at 2.307 and 0.149 keV, respectively [58]. It should be noted that the semi-quantitative evaluation is restricted to a depth of penetration equivalent to the incident electron’s wavelength, as well as a relative fraction with inadequate detection sensitivities [13]. The Cu(II) adsorption clearly shows a definite peaks of K_α_ Cu signal (at ~8.040 keV) and faint peak of L_α_ at ~0.930 KeV, respectively, indicating the existence of Cu(II) with an estimated weight percent reflecting efficient adsorption.

From the obtained results, it is clear that Cl atoms at the terminal end of activated magnetic nanohybrid (which consider the only main site for further functionalization) completely disappeared after functionalization with polyamine and amino acid, indicating that the grafting efficiency was more than 99%, and consequently the purity of the material should be like that seen in the XPS and EDX analysis of the final products adsorbent (see Figure 5, Figures S3 and S4). Moreover, the high purity of all used reagents was high purity, and all products were extensively washed to remove unreacted species.

### 3.3. Adsorption Investigations

#### 3.3.1. Effect of Initial pH

**Figure 6:**
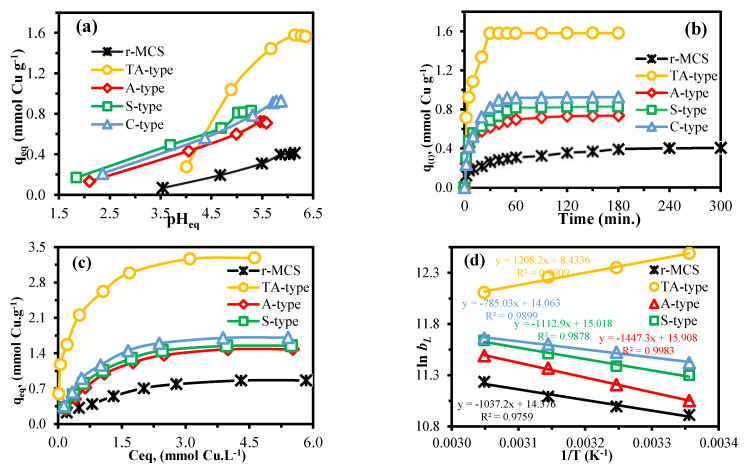
The pH effect (**a**), adsorption kinetics (**b**), isotherms (**c**), and thermodynamics (**d**).

The solution pH, as a significant physiochemical property, is crucial and has implications in the metal sorption. This parameter can alter the adsorbent’s surface charge, as well as the metal’s speciation [4,6,25]. Cu(II) adsorption was investigated throughout a pH_0_ range of 2–7 to determine an appropriate pH range for adsorption. The pH value had a substantial influence on the adsorption performance of the r-MCS, TA-type, A-type, S-type, and C-type (Figure 6a), and the capacities trend versus pH (as a function of equilibrium pH (pH_eq_)) was also quite comparable. Figure 6a shows that the adsorption capacity rose initially and subsequently nearly fixed as the initial (pH_0_: from 2.0 to 7.0) and equilibrium pH increased or climbed. The optimal pH_0_ for the r-MCS, TA-type, A-type, S-type, and C-type was 5.0–7.0, and their adsorption capacities were 0.41, 1.56, 0.71, 0.83, and 0.93 mmol Cu.g^−1^, respectively. The adsorption capabilities of the multifunctionalized materials were all greater than adsorption capacity of r-MCS (q_eq_: 0.41 mmol Cu.g^−1^) at optimal pH_0_ = 5.5–7.0. Adsorption capabilities are quite low at pH_0_ < 5.0, and rising pH equals greater capacities. In a low-pH environment, H^+^ would biasedly bind to the functional groups located on the nanohybrids surface, making it harder for Cu(II) ions to bind. When the pH steadily rises at pH_0_ > 5.0, the deprotonation process may cause Cu(II) to interact with functional groups, enhancing the nanohybrids’ adsorption ability [4,6].

All in all, as the pH_0_ went from 2.0 to 5.0, the adsorption increased, and then the pH rose over 5.0 and the Cu(II) adsorption remained steady. This process could be related to the precipitation since it impacts metal ion adsorption [4]. To prevent interference with the precipitation mechanism, an appropriate pH_0_ value ~5.0 was chosen for further studies. Indeed, the initial solution pH is pushed toward lower pH values: the pH_eq_ is closely similar with somewhat higher at initial pH values (pH_0_: 5.02–7.01) (Supplementary Figure S5a). However, the slight lowering is recorded as follows: for TA-type (at pH_eq_ of 6.16–6.35) > C-type (at pH_eq_ of 5.71–5.87) > A-type (at pH_eq_ of 5.46–5.58) > S-type (at pH_eq_ of 5.02–5.28); the solution appears to be buffered [38]. Supplementary Figure S5b depicts the relation between the log_10_*D* plot vs. pH_eq_ as being linear. The line slope for r-MCS, TA-type, A-type, S-type, and C-type was 0.1796, 3.228, 0.2894, 0.3652, and 0.437, respectively, and it is related to the stoichiometric exchange in conjunction with the attached metal ions in the Cu(II) ion-exchange mechanism [32,59].

#### 3.3.2. Adsorption Time Effect and Kinetic Studies

The implications of adsorption period on the adsorption capacities were investigated further to investigate the kinetic parameters (Figure 6b). Adsorption reaction may be separated into two stages: the initial stage observed substantial increases in adsorption capacity, followed by a progressive slowing until adsorbents achieved saturation after a time duration. The kinetic curve in Figure 6b shows that, during the quick initial stage (within the first 10 min), the adsorbents remove approximately 68.6%, 72.3%, 68.2%, 57.4%, and 55.1% for the TA-type, A-type, S-type, C-type, and r-MCS, respectively, of the total sorption, representing a physisorption mechanism. Furthermore, the initial slope is greatly enhanced, and this may be connected to the adsorbent sensitivity for Cu(II) ions (particularly for the TA-type, S-type, C-type, and A-type over r-MCS, constantly, with a specific surface area for the different nanohybrids). The TA-type adsorbent exhibited the fastest adsorption, followed by amino acid derivatives, when compared to the raw chitosan. It should be noted that the adsorption equilibrium times were ordered as follows: TA-type (30 min) > C-type (50 min) > S-type (60 min) > A-type (90 min) > r-MCS (240 min).

As a result of complementary adsorption, the second slower step (within 10 and 40 min) follows, exemplifying a chemisorption mechanism (through electrostatic interaction and/or chelation). Finally, equilibrium between adsorption and desorption is reached. According to textural analysis, all nanohybrid adsorbents had mesopores that were wide enough (the pore size range ~3.4–8.3 nm) to enable Cu(II) to flow through (Shannon radius for [Cu(H_2_O)_6_]^2+^ hydration is 0.62–0.94 Å [60]). (Corrected and done). This implies that Cu(II) ions can diffuse rather freely within the adsorbents’ mesoporous network, thus showing that the delayed sorption step contributes to global sorption, which is most likely related to resistance to intraparticle diffusion.

To properly understand the adsorption kinetics, time-dependent data were simulated. The kinetic behavior was modeled using PFORE and PSORE (Supplementary Table S1) [61,62,63]. The experimental adsorption kinetics were exhibited after modeling and linearization with several models, as illustrated in Supplementary Figures S6 and S7 and Table S5. The PSORE kinetic model’s correlation coefficient is closest to 1. As a result, the PSORE kinetic model may be used to explain Cu(II) adsorption. The PSORE model was founded on the concept that the adsorbent and adsorbate, i.e., between functionalized chitosan derivatives and Cu(II), are mostly chemisorption mechanisms [25,63]. Moreover, the goodness of the PSORE model was proven by approximately equal adsorption capacities (q_m,cal._ and q_m,exp._; Supplementary Table S5): the PSORE model’s overestimated values ranged from 1.7% to 4.7%, whereas the PFORE model’s underestimated values differ by 8.0–48.6%.

Supplementary Figure S7 depicts a multilinear plot (and Supplementary Table S5) that gives the values of the critical characteristics for intraparticle diffusion rate constants (K_id_). Supplementary Figure S7 shows three-stage multilinear plot. The first two main phase with greater K_id,1_ and K_id,2_ values were sorted as follows: K_id,1_ > K_id,2_, implying that the initial stage involves fast sorption at the most immediately accessible binding sites, as well as interior meso- and macropores [64]. The concentration tendency from the solution to the adsorbent therefore decreased, delaying the mass transfer of copper ions to inner reactive groups in mesopores. Finally, the K_id,3_ was almost zero in the last part, owing to adsorption–desorption equilibria, assuming that the adsorbents are almost saturated [25,33].

#### 3.3.3. Initial Cu(II) Concentration Effect

The adsorption equilibrium data as a function of q_eq_ = f(*C*_eq_) indicate how the Cu(II) ions can be divided between both the aqueous phase (*C*_eq_) and adsorbent surface (q_eq_) (under various initial concentrations and selected experimental conditions) [10,35,61]. The initial metal-ion concentration propels the adsorption process by eliminating the mass transfer resistance between the liquid solution and the adsorbent surface phase [61]. The Cu(II) adsorption was examined by varying the initial Cu(II) concentration and the temperature. According to Figure 6c, all adsorbents follow the same pattern: at low concentrations, the adsorption capacity increased as the initial and equilibrium Cu(II) concentrations increased. The isotherm diagram compares Cu(II) adsorption capacities at pH_0_ 5.0 and 298 K and demonstrates that the maximal real adsorption capabilities (q_eq_, in mmol.Cu.g^−1^) are arranged as follows: TA-type (3.29) > C-type (1.71) > S-type (1.55) > A-type (1.48) > r-MCS (0.86). The benefit of r-MCS functionalization is clear; the peak adsorption capacity is about 3.33 times greater for TA-type and nearly doubled for amino-acid derivatives (1.72–1.95 times). Furthermore, this reveals the vast superiority of the TA-type’s adsorption efficiency over that of the amino acid derivatives, which is compatible with the findings of pH influence and equilibration time.

The variation in the adsorption efficiency of the nanohybrids was assumed to be attributable to the differences in the functional and structural features of four amine and amino acid derivatives. As the polyamine–magnetic-chitosan derivative with diethylenetriamine, which is known as 2,2’-iminodiethylenetriamine [39]; meanwhile, for amino acid derivatives [36,65]: with alanine (a “simple” amino acid contains solely amino and carboxyl groups), Serine (a hydroxyl-containing amino acid) contains amino, carboxyl and hydroxyl groups [25] and cysteine (is a thiol-containing amino acid) possesses sulfhydryl, amino and carboxyl groups [38,42]: the major Cu(II) adsorption site is the amine/carboxyl group in addition to –SH/–S– or –OH/–O– groups. Moreover, this may explain why amine groups preferentially interact with alternative groups such as –SH or –OH (for cysteine and serine, respectively) and behave as coordination sites in adsorption reaction, as demonstrated by XPS analysis in the next section.

Table 1 shows a comparison with various adsorbents; however, a real comparison is impossible, owing to variances in experimental circumstances. The comparison shows that the TA-type and C-type adsorbents are the best, although the S-type and A-type adsorbents have an equivalent adsorption performance to conventional and traditional adsorbents. Despite this fact, some of the selected adsorbents had faster kinetics, such as Fe_3_O_4_/Cel-DETA [3], and N-aminorhodanine-modified chitosan hydrogel [17], within ~20 min, and greater adsorption capacities (e.g., urea calcium alginate beads functionalized with CR dye ~6.941 mmol.g^−1^ [6], Cd–metal–organic framework MOF ~6.842 mmol.g^−1^ [19], and Meso/micropore-controlled hierarchical porous carbon ~4.134 mmol.g^−1^ [21] and ethylenediamine-tetraacetic acid (EDTA) into layered double hydroxides (LDH) ~1.902 mmol.g^−1^ [66]). As a result, the TA-type and C-type appear to be more competitive adsorbents for Cu(II) recovery than the S-type and A-type. Adsorbents can be rated in terms of kinetics and adsorption capacity as follows: TA-type (30 min) >>> C-type (50 min) > S-type (60 min) > A-type (90 min) > r-MCS (240 min), which corresponds to the overall form of the fractional strategy to equilibria. Thus, all adsorbents have reasonable and good adsorption capacities and short equilibration times. Furthermore, with a pH range of 5.0–6.5, this implies that these nanohybrids adsorbents are suitable candidates for copper adsorption.

The Temkin, Langmuir, and Freundlich adsorption isotherms (Supplementary Table S1) were utilized and tested to simulate adsorption process (Supplementary Figure S8). As demonstrated in Supplementary Table S6, the correlation terms show that the Langmuir model had a greater association with the adsorption process than the Freundlich model, since the latter has a rather high correlation value (R^2^). Furthermore, the adsorption capabilities were regularly overestimated for the Langmuir model (∆q_eq_: 4.7–6.5%, 10.5–19.1%, and 10.8–20.1% for the TA-type amine derivative, amino acid derivatives, and r-MCS, respectively), whereas they were underestimated for the Freundlich model (∆q_eq_: 41.6–46.6%, 26.1–30.6%, and 36.5–45.2%, respectively). Consequently, a limited number of identical active sites are scattered throughout the adsorbent’s surface, resulting in monolayer uniform sorption. The resulting curve is close to the experimental locations, thus illustrating the Langmuir model’s ability to suit and fit the adsorption isotherms [13,23,69].

The Temkin model also provides a fair match to experimental profiles (in some situations). This concept is often related to a linear lowering in adsorption heat with growing surface saturation (in contrast to the Freundlich model, assuming logarithmic variation in adsorption heat) [23,69]. Supplementary Table S6 and Figure S8c show the Temkin model variables. The B_T_ coefficient is the Temkin isotherm constant, which is proportional to the adsorption heat. The B_T_ values for the r-MCS, A-type, S-type, and C-type increased with the temperature (contrary to the TA-type). Additionally, the energetic parameter (A_T_) yielded the same findings [23]; as previously stated as an example, consider the comparison between b_L_ and q_m_ in the Langmuir adsorption isotherm (Supplementary Table S6). Moreover, these findings agree with the thermodynamics results.

#### 3.3.4. Temperature Effect and Thermodynamic Parameters

The effects of temperature on adsorption ability were studied and compared at four varying temperatures (e.g., 298, 308, 318, and 328 K). Supplementary Figure S9 demonstrates that the adsorption capacity and affinity that are associated with the initial slope for both the r-MCS and amino acid derivatives increase with increasing temperature: endothermic copper adsorption. Although the TA-type shows a reciprocal pattern, the adsorption capacity marginally reduces as the temperature rises; that is, TA-type adsorption is exothermic (Supplementary Figure S9). Furthermore, the Langmuir characteristic (*b*_L_) is related to the adsorption energy: the greater the *b*_L_-value is, the greater the adsorption energy and interaction of adsorbent–adsorbate [70]. Supplementary Table S7 demonstrates that, for amino-acid derivatives, the *b*_L_ values (in L mmol^−1^) rise with temperature from 298 to 328 ±2 K and are ordered as follows: C-type (1.642–2.089) > S-type (1.454–2.042) > A-type (1.134–1.759) > r-MCS (0.988–1.360); thus, the copper adsorption is endothermic. Meanwhile, the TA-type exhibits a completely distinct reciprocal behavior: with the increasing temperature, the *b*_L_ values fall from 4.770 to 3.257 L mmol^−1^; hence, the process is exothermic.

Supplementary Table S6 reports the modeling of the adsorption isotherms; the Langmuir models match the experimental profiles only slightly better. The thermodynamics might be investigated according to the Langmuir isotherm. Lima et al. state that the Langmuir’s affinity coefficient (b_L_) characteristics are utilized after converting (to L mol^−1^ from L mg^−1^) and correcting for water molality [71]. The Van’t Hoff equation and Gibbs function equation (Equations (11) and (12)) were utilized to compute thermodynamic parameters (ΔH^o^, (the enthalpy change, in kJ mol^−1^), ΔS^o^ (the entropy change, in J mol^−1^ K^−1^), and ΔG^o^ (the Gibbs free energy change, in kJ mol^−1^)) [35,38]:

(11) $$\ln b_{L\;}=-\frac{∆H^{0}}{R}\times \frac{1}{T}+\frac{∆S^{0}}{R}$$

(12) $$∆G^{0}=∆H^{0}-T∆S^{0}$$

where the global gas constant is R = 8.314 J/mol.K, and the absolute temperature in Kelvin is T, in K. The slope and intercept of the relation diagram of ln *b*_L_ vs. 1/T plot (Figure 6d) are used to obtain the ∆H^o^, and ∆S^o^ values. Figure 6d depicts two opposite trends obtained with the amino acid and amine derivatives with increasing the temperature.

Supplementary Table S7 shows the different three thermodynamic parameters derived using the Van’t Hoff formula and the Gibbs function equation. For the amino acid derivatives, the positive ∆H° indicates that the Cu(II) adsorption is endothermic in nature [72], whilst the TA-type adsorbent has an entirely different behavior: the negative value indicates the exothermic nature [59,73]. Moreover, the ∆H^o^ values < 40 kJ/mol demonstrate the presence of physical forces in the Cu(II) adsorption reaction. The net ∆H^o^ is the summation of the dehydration enthalpy (∆H_dehydr_, that is thought to be positive because of the energy necessary to break the hydrated metal ions’ ion–water and water–water bonds) and the enthalpy of complexation (∆H_complex_, also negative) [47]. Furthermore, the endothermic process includes several phases, namely Cu(II) transfer to the solid surface, dehydration, and complex formation [74]. All of these reactions require heat to induce the adsorption (reciprocal to the TA-type).

The positive ∆S^o^ revealed that adsorption improves the randomness of the state environment and global system and that the solution grew more disordered [23,37]. Moreover, the negative ΔG° values within a comparable range show that the sorption process is spontaneous. According to Zai [75], sorption is both physical and chemical in nature whenever the ΔG° values ranges from −20 to −80 kJ mol^−1^. Moreover, Supplementary Table S7 indicates the absolute values (│ΔH°│< │TΔS°│); the copper adsorption is regulated by entropic rather than enthalpic changes [26,35].

#### 3.3.5. Adsorbent Reusability

Adsorption/desorption cycles up to six runs were carried out. The elution of Cu(II) was performed using thiourea (0.25 mol.L^−1^) acidified with H_2_SO_4_ (at pH 2). The experimental adsorption and desorption conditions are systematically listed under Supplementary Table S8. Six sequential sorption/desorption cycles are shown in Supplementary Table S8, with a little decline in both adsorption and desorption efficiency for each sorption stage. After the sixth cycle, the efficiency losses in adsorption and desorption were about 5.3–9.1% and 4.5–5.3%, respectively. This confirmed that all adsorbents exhibited high levels of stability, reusability, and durability for repeated use.

#### 3.3.6. Chemical Stability Examination

The nanohybrid samples were soaked and agitated for one day in various pH solutions, ranging from pH_0_ 1 to 10, in the pH_ZPC_ experiment. After collection and magnetic separation, the nanohybrids were constant and very stable. The dry samples’ mass-loss efficiencies after acidic and alkaline treatment against the mass of the untreated samples (under identical conditions) were ~1.9–4.7%. The maximal mass loss values were recorded and observed at pH_0_: 1.0 > pH_0_: 2.0 because of the surficial Fe_3_O_4_ particles’ dissolving [25]. Moreover, after the sixth adsorption/desorption cycle, C-type and TA-type FTIR spectra (as examples of nanohybrids) confirmed the functional stability and chemical resistivity (see Supplementary Table S2). Moreover, the little drop in adsorption/desorption characteristics supports and verifies the durability of all the nanohybrid materials [26]. These findings showed how extremely robust and durable these materials are.

#### 3.3.7. Metal Adsorption Interaction and Complexation

**Figure 7:**
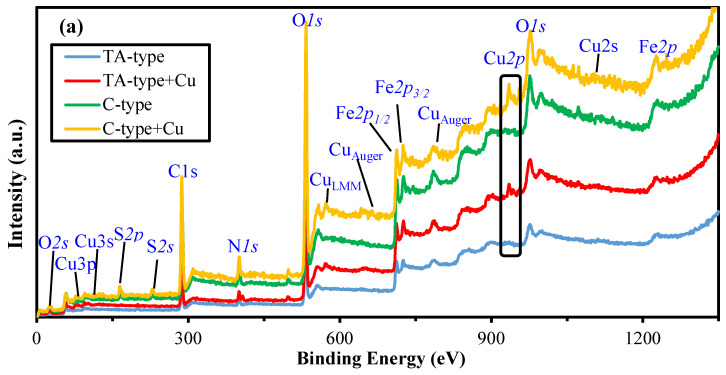

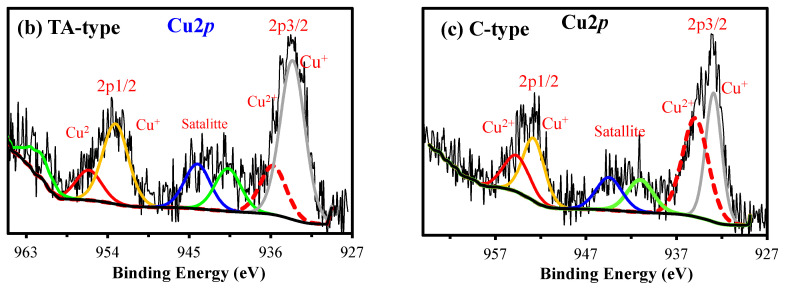
XPS survey spectra of raw TA-type and C-type adsorbent before and after Cu(II) adsorption (**a**) and core level for Cu2p (**b**,**c**).

XPS spectroscopy. XPS was employed to investigate the chemical environments of surface components, as well as the composition features of the survey XPS spectrum of TA-type and C-type (as a representative for amino acids-derivatives) before and after Cu(II) sorption (Figure 7). The core level signals of Cu2*p* are quite intense for TA-type and C-type adsorbents (see Figure 4). Cu(II) binding is underlined by the formation of a doublet at ~932.98–943.08 eV (Cu2*p*_3/2_) and ~952.18–953.58 eV (Cu 2*p*_1/2_).

Supplementary Table S3 outlines the band-fitting results from HRES spectra for the bare TA-type and C-type monohybrids that were considerably compared after Cu(II) binding for C1*s*, O2*s*, N1*s*, S2*p*, Fe2*p*, and Cu2*p* signals (Figure 7a). Notably, the most substantial changes are detected for the remaining chosen signals of the main elements, where Fe2*p* was hardly affected after Cu(II) adsorption (see Figure 3). Variations in the proportional contributions and change in BEs of the various bands provide the active sites engaged in the adsorption process that are mostly preceded by Cu(II) interacting with N–, S–, and O-containing groups such as (amine >NH/–NH_2_), (–SH/thiolate), and (–OH/>C=O/–COO^−^), respectively. The new significant Cu2*p* double peaks in the XPS spectrum clearly verified the Cu(II) adsorption by TA-type and C-type. The Cu2*p* excitations can be deconvoluted into some multiple-Cu2*p* peaks into two subpeaks’ spin–orbit (L–S) split assigned to Cu2*p*_3/2_ (~953 eV) and Cu2p_1/2_ (~932 eV), accompanied by the corresponding ~8–9 eV satellite peaks from the Cu2*p*_3/2_ (Figure 7b,c) [8]. These satellite peaks can be assigned as 3d→4s shake-up transitions [76].

The deconvolution of the Cu2p_3/2_ displays two different BEs states that can be ascribed to CuO and cupric ions that reside in octahedral positions and interact strongly with both adsorbents [12], respectively. The two main large asymmetric peaks were fitted into two subpeaks for Cu^+^ 2*p*_3/2_ (at 932.70–933.38 eV), Cu^2+^ 2*p*_3/2_ (at 934.73–935.52 eV), Cu^+^ 2*p*_1/2_ (at 945.26–952.94 eV), and Cu^2+^ 2*p*_1/2_ (at 952.56–955.89 eV) which were situated (Supplementary Table S3) [8]. The Cu^+^ 2*p*_3/2_ at 932.70–933.38 eV, and Cu^+^ 2*p*_1/2_ at 945.26–952.94 eV were observed, owing to low values of the shake-up satellite/main peak ratio, likely related to a reduction in Cu^+^ [12].

In light of the mechanism discussion, various hypotheses for the coordination mechanism during adsorption reactions have been offered. Interaction mechanisms of Cu(II) with TA-type and C-type adsorbents were suggested from the FTIR and XPS findings. The coordination number for Cu(II) is 4–6. Cu(II) with six coordination number having d^9^ configuration favors a distorted octahedral structure that is governed by a direct result of Jahn–Teller instabilities [77].

Based on XPS analyses, Cu(II) may exist in three different forms (Cu(II), [Cu(NO_3_)]^+^, and [Cu(OH)]^+^), where NO_3_^−^ group from standard Cu(NO_3_)_2_ solution. Scheme 4 depicts the intuitive graph. Thus, two contribution styles (I and II) form chelating rings with five members; more chelate rings enhance the stability of the formed complex and subsequently improve the metal adsorption capability. Furthermore, Scheme 4 displays the hypothesized desorption mechanism (via employing acidified thiourea), since the solution’s acidity promotes and breaks the chelation modes (model(V)). Moreover, thiourea is structurally identical to urea, with the exception that the oxygen atom is substituted with a sulfur atom. Thus, the CS(NH_2_)_2_ molecule may bind and coordinate with both terminal N atoms of –NH_2_.

#### 3.3.8. Adsorption Selectivity

**Figure 8:**
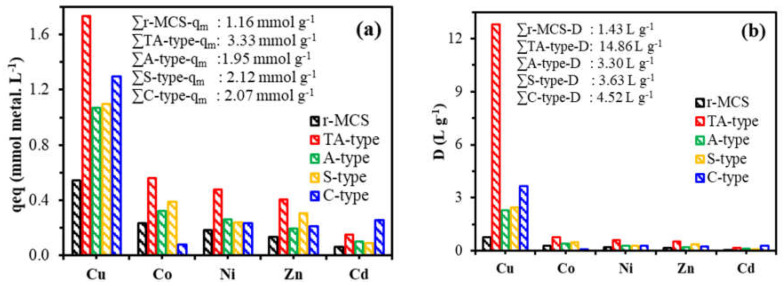

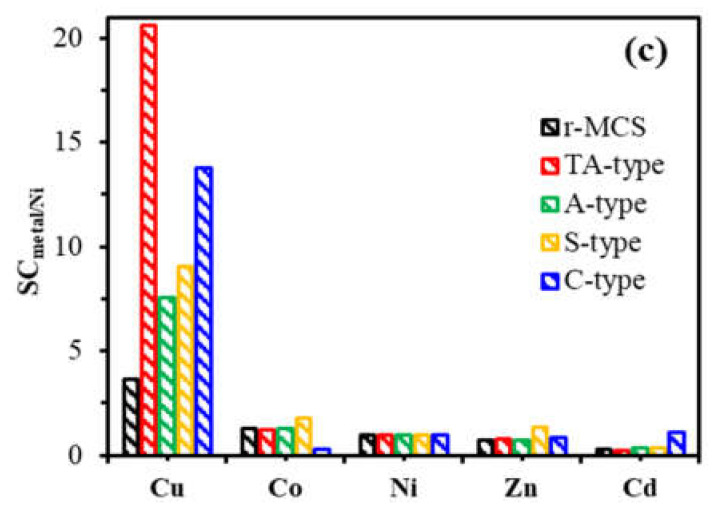
Selectivity experiments through sorption capacities (**a**), distribution coefficients (**b**), and selectivity coefficient (SC_metal/Ni_) (**c**): (pH_0_, 5.0; SD, 0.5 g.L^−1^; T, 298 K; time, 120 min; 200 rpm).

To assess the efficacy of such nanohybrids adsorbents for Cu(II) removal and adsorption, the adsorption behavior of Cu(II) from equimolar solutions of the multicomponent system (C_0_: 1 mmol.L^−1^ of divalent metal ions, e.g., Cd(II), Zn(II), Co(II), and Ni(II)), with a total concentration that reaches up to 5.01 mmol metal.L^−1^ at pH_0_ 5.0 and room temperature 25 °C. For r-MCS and nanohybrid derivatives, the maximum adsorption capacity (qm, mmol.metal.g^−1^), distribution ratios (D, L.g^−1^), and selectivity coefficients (SC_metal/Ni_ = D_metal_/D_Ni_, where Ni(II) was chosen as the standard because of its lower adsorption capacity), are shown in Figure 8. The cumulative adsorption capacities are ordered as follows (q_m_, in mmol.g^−1^): TA-type (∑q_m_: 3.33) > S-type (∑q_m_: 2.12) > C-type (∑q_m_: 2.07) > A-type (∑q_m_: 1.95) > r-MCS (∑q_m_: 1.16). These q_m_-values were higher than those for Cu(II) in single solutions by 1.09%, 35.80%, 20.47%, 31.85%, and 30.79% for TA-type, S-type, C-type, A-type, and r-MCS, respectively. The enhanced accessibility of multi-central amine, hydroxyl, and thiol groups for metal ion adsorption and binding might explain the lack of selectivity for Cu(II). Copper adsorption capacities for TA-type, C-type, S-type, A-type, and r-MCS were 1.73, 1.29, 1.10, 1.07, and 0.58 mmol Cu.g^−1^, respectively, and were lower than those of synthetic solutions. This suggests that all nanohybrid adsorbents retained a strong affinity, adsorption capacities, and selectivity for Cu(II) over other co-elements, despite a complex composition containing high salty and competing metals. This might be due to a differential in reactivity to other co-ions. This effect is most likely explained by the ionic radius differences and changes around 0.69–0.95 Å for Cd(II), Co(II), Zn(II), Ni(II), and ~0.75 Å for Cu(II). This difference in size provides more adaptability to reactive group accessibility, which could be impacted by steric hindrance.

Moreover, the C-type adsorbent is considerably more efficient and consistently superior for Cu(II) adsorption than other adsorbents. The second affinity chosen is Cd(II); this is most probably due to sulfur functional groups (i.e., regarded as a soft base), which are more reactive and sensitive to soft acids, such as Cd(II) [78], lowering the total affinity and selectivity of C-type adsorbent for Cu(II). Generally, no obvious association exists between HSAB (hard and soft acid-and-base theory) rank and metal adsorption affinity; Cd(II) is regarded as a soft acid. Other co-metals, on the other hand, are borderline metals (e.g., Ni(II), Zn(II), Co(II), and Cu(II)) [78].

The EDX measurements after multicomponent treatment reveal high selective Cu(II) adsorption for all materials (Figure 5 and Supplementary Figure S4). The other co-ions of multi-elements were identified by two characteristic peaks of Kα and poor Lα signals as follow: at 8.637 keV and 1.012 keV, respectively, for Zn signals; at 7.477 keV and 0.851 keV, respectively, for Ni signals; and at 6.929 keV and 0.776 keV, respectively, for Co signals. Meanwhile, there was the appearance of Cd signals of Kα at 23.175 keV and Lα at 3.133 keV [58].

These selectivity patterns were reflected in the D (distribution ratio) plots, which emphasize the increased affinity for Cu(II) in particular (Figure 8b). Therefore, Cu(II) showed highest D-values in L.g^−1^ with the following orders: TA-type (D: 12.79) > C-type (D:3.65) >S-type (D: 2.43) > A-type (D: 2.29) > r-MCS (D: 0.81). Moreover, the graph of selectivity coefficients versus Ni(II) (i.e., SC_metal/Ni_) presented in Figure 8c can be used to sort the metal ions’ selectivity for each adsorbent:

TA-type: Cu(II) (20.58) >>> Co(II) (1.25) > Ni(II) (1.00) > Zn(II) (0.82) > Cd(II) (0.26).

C-type: Cu(II) (13.77) >>> Cd(II) (1.13) > Ni(II) (1.00) > Zn(II) (0.88) > Co(II) (0.31).

S-type: Cu(II) (9.04) >> Co(II) (1.78) > Zn(II) (1.39) > Ni(II) (1.00) > Cd(II) (0.36).

A-type: Cu(II) (7.59) >> Co(II) (1.28) > Ni(II) (1.00) >Zn(II) (0.72) > Cd(II) (0.35).

r-MCS: Cu(II) (4.12)> Co(II) (1.30) > Ni(II) (1.00) >Zn(II) (0.71) > Cd(II) (0.29).

The results show that the adsorbents have a high and strong affinity and selectivity for copper ions over other co-metals. In a trial to interpret these adsorption patterns via the physicochemical parameters of different co-metal cations, the concepts of QSAR were applied [79]; some key characteristics include hydrated ionic radius (in Aº), electronegativity (Pauling units), and effective ionic charge (Supplementary Table S9 and Figure S10). The statistical data analysis (through R^2^ values) showed skewing with the grouped co-cations (Cd(II), Zn(II), Co(II), and Ni(II)) versus Cu(II). The highest correlation coefficients were achieved when a direct relationship between adsorption capacity versus effective ionic charge (i.e., the most effective factor) and followed by atomic number (i.e., the second one) (Figure S10), except for the C-type, which may be controlled by HSAB rather than QSAR. 

#### 3.3.9. Direction of a Relationship between Variables

Meanwhile, there is not a strong relation between the QSAR tools and/or HSAB ranking and the obtained metal adsorption order.

### 3.4. Simulation and Graphical Mathematical Modeling

Recent multimedia advancements have expanded in the 3D (three-dimensional) information available. Therefore, effective data management involves appropriate approaches for its representation and processing. Nonlinear models have lately grown in popularity, not just for accuracy criteria but also for broadening the model’s applicability [59,80]. The system requirements are effectively realized due to the focus on correctness throughout the simulation study. The latter is performed by the use of MATLAB software, a nonlinear regression, and a graphical technique. To measure the fitting and goodness of the suggested technique, the description evaluates the broad distribution of interactions between particular points (pH_0_ and C_0_ versus their response q_eq_). The general Langmuir equation in nonlinear form (q = q_m_.KC^n^/(1 + KC)^m^) was utilized to illustrate multiple 3D models [61,80] because the Langmuir isotherm matches the data better.

Both initial pH and Cu(II) concentration parameters versus their response Cu(II) adsorption can be connected by Equation (13) [80]. By adapting the Langmuir equation to integrate the initial pH and Cu(II) concentrations, we obtained the general formula and shown in Equation (13). It was adapted using ΔG (Gibbs energy change), as shown below in Equation (14):

(13) $$\mathrm{For}\;q_{\left({\mathrm{pH}}_{0\;},C_{0}\right)}=\frac{q_{m}\times k\times {\mathrm{pH}}^{m}\times C^{n}}{{\left(1+k\times {\mathrm{pH}}^{f}\times C^{e}\right)}^{g}}$$

(14) $$∆G=-R\times T\times {\mathrm{loge}}^{k}=\frac{-8.314\times 298\times \log_{e}^{\left(4.166\times 63.546\times 1000\right)}}{1000}=-30.9361\;$$

where q_e(pH0,*C*0)_ is the resultant adsorption capacity for a given pH_0_, C_0_, and q_m_, the maximum adsorption capacity (for TA-type and A-type was 3.29 and 1.48 mmol Cu.g^−1^, respectively). *C*_0_ and pH_0_ refer to initial Cu(II) concentration and initial pH, respectively [80]. *K* is the adjusted Langmuir constant. Letter symbols (e.g., m, n, f, e, and g) correspond to constant factors of the valuables.

**Figure 9:**
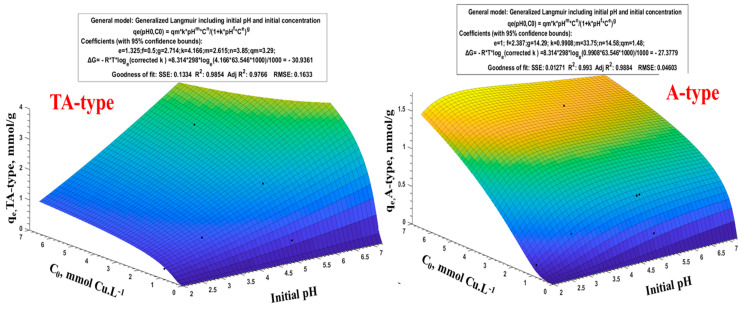
Generalized Langmuir model, including Cu(II) adsorption (q_e_) vs. initial pH and *C*_0_.

Depending on the adsorption results at the various pH_0_s and C_0_s, in line with the methodical process in Equation (13), the experimental results were visually displayed in a 3D graph (Figure 9 and Supplementary Figure S11). This equation can be used for the pH_0_ (range from 2.0 to 7.0) and C_0_ (range from 0.32 to 6.27 mmol Cu.L^−1^); these ranges suit the equation well. This mathematical formula often correlates better with experimental profiles: SSE, R^2^, and Adj R^2^ components support this fitting [80]. Furthermore, the calculated free energy (ΔG: −30.94 kJ.mol^−1^ at 298 K) is generally comparable with practical thermodynamic findings (see Supplementary Table S10).

Figure 9 and Supplementary Figure S11 (and Equation (13)) illustrate how the function power is and present all parameters. The greater the order of exponential magnitude, the greater the effect on the influence and the response of q_e_(pH_0,_*C*_0_) [59]. According to the results of Equation (13)’s application, the numerical values of the exponential for all adsorbents are presented in Supplementary Table S10. For TA-type, the exponentials are ordered and arranged as follows: n = 3.85 > m = 2.615 > g = 2.714 > e = 1.325 > f = 0.50. Based on n and m values of *C*^n^ and pH^m^, respectively, the most crucial element is the initial concentration (*C*_0_), followed by the second component (pH_0_). Moreover, this relates to the exponent values of the denominator’s coefficients, i.e., e and f values for *C*_0_ and pH_0_, respectively, whilst the product of both (pH_0_×*C*_0_) is more effective than *C*_0_ and pH_0_ individually. This is congruent with the experimental findings.

The extent to which the theoretical findings coincide with the experimental data via the error percentage is used (Error % = (q_e,exp._ − q_e,Math._)×100)/q_e,exp._) to validate the mathematical methodology. Supplementary Table S11 illustrates the validation and comparative findings of the mathematical modeling for the pH_0_ range (2.0 to 7.0) and initial concentration ranges (*C*_0_: 0.32–6.27 mmol Cu.L^−1^). The mathematical method’s accuracy (Δ) is assessed and found to be overstated by (∆ < 7.78%) and underestimated by (∆ < 3.51%). The presence of a blank gap on the figure’s surface in Figure 9 and Supplementary Figure S11 indicates that the results, in this case, cannot be applicable to the TA-type. Moreover, Supplementary Table S11 shows that the high error percent (Error %) for TA-type and other adsorbents was observed at pH_0_ 2.0 (except for A-type), and also at pH_0_ 7.0 for TA-type only.

## 4. Conclusions

Four eco-friendly superparamagnetic multifunctionalized chitosan nanohybrids were prepared suitably to be used as efficient adsorbents for Cu(II) ions. A facile method for synthesizing superparamagnetic Fe_3_O_4_/chitosan nanohybrids was reported via a heterogeneous nucleating of Fe^(II)/(III)^ in a chitosan solution. The grafting of diethylenetriamine, alanine, serine, and cysteine moieties (via an intermediary chlorinated step of crosslinked epichlorohydrin–chitosan/Fe_3_O_4_ nanohybrid), giving TA-type, A-type, S-type, and C-type, respectively, improves the adsorption capacities (vs. non-functionalized chitosan nanohybrid) due to the specific reactivity of amine, carboxylate, hydroxyl, and sulfhydryl (thiol and/or thioether group) groups. The chemical changes are validated by CHNS, TEM, pH-titration, XPS, VSM, XRD, and FTIR analysis. The grafting of additional active sites considerably improves Cu(II) adsorption properties: the maximal adsorption capacity is more than triplicated (~3.33 times) for polyamine and nearly doubled for amino acid derivatives (~1.73–1.96 times) when compared to raw chitosan nanohybrid at pH_0_ ~5.0. The equilibrium is achieved after 30–90 min of contact and matches the pseudo-second-order rate equation well. The Langmuir equation efficiently fits the sorption isotherms, and the Cu(II) adsorption process is endothermic for the amino acid derivatives (opposite to the amine derivative), spontaneous, and followed by increasing the randomness in the system. Copper desorption is exceedingly efficient (using 0.25 mol.L^−1^ thiourea solutions at pH 2.0), and the nanohybrid could be recycled at least six times. All nanohybrids materials display outstanding functionality, durability, performance, strength, and stability. In the selectivity test, copper adsorption from equimolar multicomponent systems demonstrates that all adsorbents have a surprising affinity toward Cu(II). The affinities are compatible with the metals’ HSAB properties and the adsorbents softer/harder ordering. Furthermore, QSAR methods were employed to correlate intrinsic co-ion characteristics with their comparative affinity. Moreover, a mathematical simulator is being developed and built based on experimental data. The mathematical formula was validated and confirmed with measured values and the output findings.

The future perspectives of the presented research are as follows: this work can be tested for a wide range of heavy metal ions and cationic and anionic dye removal due to the wide effective pH range, high stability, and multifunctionalization with different active sites; thus, the developed chitosan hybrid in this study is a promising material in the field of wastewater treatment.

## Figures and Tables

**Table 1:** Copper adsorption performances using different adsorbents.

Adsorbent	Operating Condition (pH_0_, Time, Temp. (in °C), SD (g.L^−1^))	q_m_, mmol Cu.g^−1^	Ref.
Pristine natural zeolite	5.5, 24 h, 26 °C, 1.0	0.235	[28]
PDA treated zeolite powders		0.45	
Zr-based MOFs (MOF-801)	5.5, 180 min, 27 °C, 0.4	0.278	[16]
DTPA modified sludge	3.0, 60 min, 25 °C, 0.4	0.557	[67]
Foamed geopolymer sphere	5.0, 48 h, 27 °C, 1.0	0.596	[68]
CS-GLA	5.0, 360 min, 26 °C, 5.0	0.601	[17]
Aminorhodanin@CS	5.0, 20 min, 26 °C, 5.0	0.984	
Melamine–HCHO–DTPA	4.5, 60 min, 20 °C, 10.0	0.729	[7]
N-Ch-Sal	5.0, 120 min, 26 °C, 1.0	1.331	[10]
Mag. Cel-DETA (MCGT)	5.4, 5 min, 25 °C, 1.0	1.449	[18]
Silkworm excrement biochar	5.0, 24 h, 25 °C, 1.0	1.471	[4]
MgAl-EDTA-LDH	5.0, 60 min, 25 °C, 1.25	1.901	[66]
Meso/microporous Carbon	5.0, 120 min, 25 °C, 0.35	4.134	[21]
Cd-terephthalate-MOF-2	6.0, 60 min, 27 °C, 0.5	6.842	[19]
CR dye@Urea Ca-alginate	6.5, 90 min, 55 °C, 1.5	6.94	[6]
r-MCS	5.0, 240 min, 55 °C, 0.5	0.987	*Here*
A-type	5.0, 90 min, 55 °C, 0.5	1.696	
S-typr	5.0, 60 min, 55 °C, 0.5	1.745	
C-type	5.0, 50 min, 55 °C, 0.5	1.921	
TA-type	5.0, 30 min, 25 °C, 0.5	3.287	

## Data Availability

All data generated or analyzed during this study are included in this article.

## Supplementary Materials

The following supporting information can be downloaded at: https://www.mdpi.com/article/10.3390/polym15051157/s1, Table S1. Selected kinetics and adsorption isotherms models. Table S2. Effect of functionality on the adsorbent feature and adsorption characteristics. Table S3. Analysis of XPS spectra: assignment of the core-level signals (BEs and AF). Table S4. FTIR chemical assignments. Table S5. adsorption kinetics parameters of Cu(II) adsorption. Table S6. Adsorption isotherms parameters of Cu(II) adsorption. Table S7. Thermodynamic parameters for Cu(II) adsorption. Table S8. Metal desorption and adsorption cycles. Table S9. Chemical properties of selected metal ions. Table S10. The parameters and constants of Generalized Langmuir equation and Goodness of fit for all adsorbents. Table S11. Comparison for experimental adsorption capacity (q_e,exp_) and mathematically calculated (q_e,cal._) for r-MCS, TA-type, A-type, S-type, and C-type adsorbents. Figure S1. Textural characterization of TA-type (a), S-type, A-type (b), and C-type (c): BET surface area analysis and pore size analysis (insert). Figure S2. Thermogravimetric analysis of S-type nanohybrid: TGA (a); DTG (b); magnetization curves (c); and the pH_ZPC_ of r-CS, MCS, and all nanohybrids (d). Figure S3. XPS survey spectra of TA-type, A-type, S-type, and C-type nanocomposites. Figure S4. EDX measurements of raw r-MCS, A-type, and S-type materials before (a) and after Cu(II) adsorption from synthetic solution (b) and mixed system(c). Figure S5. Effect of initial pH versus equilibrium pH (a) and plot of distribution ratio (D, L g^−1^) vs. pHeq (b). Figure S6. Kinetics models PFORE and PSORE for Cu(II) adsorption. Figure S7. Kinetics models of interparticle diffusion for Cu(II) sorption. Figure S8. (a) Langmuir isotherm models, (b) Freundlich isotherm models, and (c) Temkin isotherm models for Cu(II) adsorption. Figure S9. Adsorption isotherms for all adsorbent types at different temperatures (i.e., T: 298, 308, 318, and 328 K); time of 120 min for TA-, A-, S-, and C-type; and time of 240 min for r-MCS). Figure S10. Correlation between intrinsic physicochemical characteristics (atomic number (a), hydrated ionic radius (b), electronegativity (c), and effective ionic charge (d)) and their adsorption capacities (q_m_). Figure S11. Generalized Langmuir including initial pH and initial Cu(II) concentration. References [23,25,30,31,32,34,38,39,43,53,54,61] are cited in Supplementary Materials.
