# Supplementary material for: Superparamagnetic Multifunctionalized Chitosan Nanohybrids for Efficient Copper Adsorption: Comparative Performance, Stability, and Mechanism Insights
Source: Polymers (Basel). 2023 Feb 24;15(5):1157. doi: 10.3390/polym15051157 (PMC10007229; doi:10.3390/polym15051157)
Supplement: Supplementary file 1 [file polymers-15-01157-s001.zip › polymers-2205186-supplementary.pdf]

Supporting Information

Superparamagnetic Multifunctionalized-Chitosan Nanohybrids for Efficient Copper adsorption: Comparative Performance, Stability, and Mechanism Insights

Ahmed A. Al-Ghamdi [1], Ahmed A. Galhoum [2], Ahmed Alshahrie [1,3], Yusuf A. Al-Turki [4,5], Amal M. Al-Amri [6] and S. Wageh [1,*]

1 Department of Physics, Faculty of Science, King Abdulaziz University, Jeddah 21589, Saudi Arabia

2 Nuclear Materials Authority, El-Maadi, Cairo P.O. Box 530, Egypt

3 Centre of Nanotechnology, King Abdulaziz University, Jeddah 21589, Saudi Arabia

4 Department of Electrical and Computer Engineering, Faculty of Engineering, King Abdulaziz University, Jeddah 21589, Saudi Arabia

5 K. A. CARE Energy Research and Innovation Center, King Abdulaziz University, Jeddah 21589, Saudi Arabia

6 Physics Department, Rabigh College of Science and Arts, King Abdulaziz University, P.O. Box 344, Rabigh 21911, Saudi Arabia

* Correspondence: wswelm@kau.edu.sa

Section S1 – Material and methods

S1.1. Characterization

(C, H, and N) was determined using an automatic analyzer (CHNS Vario EL III-elementar analyzer, Elementar, Germany). A Philips X-ray generator type PW 3710/31, Cu Kα (λ=1.544) (Philips, Japan) was used to measure X-ray diffraction. The FTIR spectra were analyzed by a Thermo-Fisher Nicolet IS10 spectrometer (Waltham, MA, USA). The morphology and size distribution were analyzed by TEM-JEOL-2100 transmission electron microscope (JOEL, Tokyo, Japan). The magnetic characteristics were determined using a vibrating sample magnetometer (VSM; 730T, Lakeshoper, USA). The energy-dispersive X-ray spectroscopy (EDX, a ESEM model Philips XL 30, Eindhoven, Netherlands) was used to assess the chemical analysis. A Quantachrome NOVA 3200 (Boynton Beach, FL, USA) analyzer was used to measure the surface area using N_2-adsorption isotherms under a degassing temperature of 40 ^{o}C for 12 hours, while the pore volume was measured according to the BJH method. The K-Alpha spectrometer (Thermo Fisher Scientific, Waltham, USA) was used to acquire X-ray photoelectron spectroscopy (XPS) data using a monochromatic Al Kα-radiation source (200 W). The sorbent's pH zero-point of charge (pH_{ZPC}) was measured using the pH-drift method [1, 2]: the sorbents were equilibrated for 48 h under agitation with a series of 0.1 M NaCl solutions with different initial pH-values (pH_0); the equilibrium pH (pH_{eq}) was recorded and the pH_{ZPC} is the pH at which ΔpH =0.

Table S1. Selected kinetics and adsorption isotherms models [3, 4].

Process	Model	Equation	Parameters	
Kinetics	PFORE	$q(t) = q_{eq,1}(1 - e^{k_1 t})$	$q_{eq,1}$ (mmol g^{-1})	k_1 (min^{-1})
	PSORE	$q(t) = \frac{q_{eq,2}^2 \times k_2 \times t}{1 + q_{eq,2} \times k_2 \times t}$	$q_{eq,2}$ (mmol g^{-1})	k_2 (L $mmol^{-1}$ min^{-1})
	sRIDE (Weber & Morris)	$q_{(t)} = k_{int.,i}.t^{0.5} + C$ Several linear sections corresponding to different regimes of resistance (i) to intraparticle diffusion may co-exist ($K_{int, i}$) (linear regression calculation)	$K_{int.,i}$ (mmol g^{-1} $min^{-0.5}$)	
Isotherms	Langmuir	$\frac{C_{eq}}{q_{eq}} = \frac{C_{eq}}{q_{max}} + \frac{1}{bq_{max}}$	q_{max} (mmol g^{-1})	b_L (L $mmol^{-1}$)
	Freundlich	$q = k_F C_{eq}^{1/n}$	k_F	N (dimensionless)
	Temkin	$q_{eq} = B_T lnC_{eq} + B_T lnA_T$ Where, $B_T = \frac{RT}{b_T}$	A_T (L $mmol^{-1}$)	b_T (J mol^{-1})

Table S2. Effect of functionality on the adsorbent feature and adsorption characteristics.

Properties	rCS	MCS	CMCS	ACMCS	TA-type	A-type	S-type	C-type
Magnetic saturation (Ms, emu/g)	-	42.966	-	-	31.134	26.944	23.206	22.837
Coercive field (H_C)	-	57.166	-	-	22.694	24.609	46.112	39.811
Remanence (M_r)	-	0.721	-	-	0.578	0.271	0.897	0.754
Calculated particle size: From TEM (nm)	-	-	-	-	8.45 ±1.4	10.43 ±1.9	14.79 ±3.2	14.01 ±3.7
: From XRD (nm)	-	-	-	-	9.01	9.43	10.83	9.78
C, (%)	39.99	18.91	18.35	15.82	18.34	17.48	16.59	20.51
H, (%)	6.88	4.07	3.93	3.23	3.76	2.93	2.87	4.01
N (%)	7.79	3.58	3.06	1.98	5.21	3.77	3.62	3.29
N (mmol g^{-1})	5.56	2.56	2.18	1.41	3.72	2.69	2.58	2.35
S, (%)	-	-	-	-	-	-	-	3.01
Specific surface area (S_{BET}, m^2 g^{-1})	-	-	-	-	75.27	60.382	61.052	42.56
V_P (cm^3 STP g^{-1})	-	-	-	-	0.5696	0.3598	0.4105	0.1134
Mean pore diameter (nm)	-	-	-	-	27.533	22.466	26.997	3.839
pH_{ZPC}	8.57 ±0.02	7.26 ±0.03	-	-	8.48 ±0.02	7.88 ±0.01	7.66 ±0.02	7.78 ±0.03
Optimum pH_0	-	5.0-6.0	-	-	5	5	5	5
Teq (min)	-	240	-	-	30	90	60	50
$q_{m,exp}$ (mmol Cu.g^{-1})@ 328 K	-	0.985	-	-	2.946	1.697	1.735	1.913
b_L (L $mmol^{-1}$) at 328 K	-	1.364	-	-	3.257	1.759	2.042	2.088
ΔH° (kJ mol^{-1})	-	8.623	-	-	-10.045	12.033	9.253	6.527
ΔS° (J mol^{-1} K^{-1})	-	119.52	-	-	70.12	132.26	124.86	116.92
The cumulative q_m (mmol g^{-1}) in complexed solution		1.163			3.329	1.954	2.122	2.073
Sensitivity in complexed solution*	-	-35.1%	-	-	-47.3%	-27.7%	-29.5%	-24.8%

Table S3. Analysis of XPS spectra: assignment of the core-level signals (BEs and AF).

Name	TA-type		TA-type+Cu		A-type		S-type		C-type		C-type+Cu		Chemical assignments
	BE (eV)	AF (%)	BE (eV)	AF (%)	BE, (eV)	AF, (%)	BE, (eV)	AF, (%)	BE (eV)	AF (%)	BE (eV)	AF (%)	
C1*s*	285.56	51.59	284.53	15.04	286.78	55.56	286.66	91.23	285.36	22.88	284.76	19.11	C-H, C-C, $C_{advent.}$
	286.23	26.66	286.03	62.82	288.09	42.42	288.16	3.04	286.37	24.32	286.05	47.37	C-OH, C-N, C-O-C, C-S
	287.49	21.75	287.66	13.48	288.25	2.02	289.22	5.74	287.23	52.8	287.55	33.51	π-π* sat., C=O (Amide)
			288.12	8.66									–COOH
N1*s*	399.12	60.32	399.52	65	400.55	51.39	399.27	33.4	399.54	64.37	399.51	60.24	C-N (amide), N-H
	399.58	39.68	401.77	9.08	400.67	48.61	400.41	66.6	401.36	35.63	401.29	25.03	$-NH_2$, >NH (amine)
			406.39	25.92							406.59	14.74	NO2 (nitrite ions)
O1*s*	529.61	30.14	529.54	14.18	530.82	26.73	529.7	11	530.17	20.29	529.99	19.09	Lattice O (Fe–O, Fe_3O_4)
	530.87	7.32	530.57	24.22	533.23	69.52	530.89	31.13	531.99	42.76	532.37	77.91	C=O, O-C=O
	532.51	62.54	532.4	61.6	534.76	3.76	533.03	57.87	532.98	36.95			C-OH, H_2O, C-O-C
											535.14	3.00	NO_2^- (nitrite ions)
Fe 2*p*	710.27	30.9	710.27	16.58	711.67	37.14	711.01	31.23	710.68	31.18	710.48	26.99	Fe $2p_{3/2}$ (Fe^{3+}–octa)
	712.81	18.42	712.16	25.87	714.19	15.37	713.59	17.67	713.23	19.42	713.02	21.19	Fe $2p_{3/2}$ (multipl–Fe^{2+}–octa)
	716.45	7.7	715.81	9.58	717.51	7.82	717.18	7.97	716.96	7.97	716.89	9.36	Fe $2p_{3/2}$ (Fe^{3+}–tetra)
	719.47	8.93	719.43	11.31	720.81	8.68	720.41	9.44	719.99	8.72	720.21	9.73	Fe $2p_{1/2}$ (Fe^{3+}–Satallite)
	723.80	19.92	724.02	20.44	725.01	18.49	724.60	19.97	724.31	19.88	724.25	20.29	Fe $2p_{1/2}$ (Fe^{2+}–octa)
	727.12	8.78	727.24	9.77	727.92	9.03	727.96	8.49	727.69	7.8	727.71	7.61	Fe $2p_{1/2}$ (Fe^{3+}–tetra)
	732.42	5.35	732.38	6.45	732.9	3.48	733	5.22	732.39	4.97	732.58	4.83	Fe $2p_{3/2}$ (Fe^{3+}–octa)
S 2*p*											163.38	25.17	C–S ($2p_{3/2}$)
									164.17	82.27	164.16	42.83	C–S ($2p_{3/2}$)
									168.05	17.73	168.04	32	C–S–C (S $2p_{1/2}$)

	933.38	35.58	932.70	23.76	Cu 2$p_{3/2}$ (Cu^{+})
	935.52	11.45	934.73	29.47	Cu 2$p_{3/2}$ (Cu^{2+})
	940.51	10.09	954.45	11.1	Cu 2$p_{3/2}$ (Cu^{+}- Satallite)
Cu *2p*	943.89	10.59	940.79	9.76	Cu 2$p_{3/2}$ (Cu^{2+}- Satallite)
	952.94	19.19	944.26	9.89	Cu 2$p_{1/2}$ (Cu^{+})
	955.89	7.23	952.56	16.02	Cu 2$p_{1/2}$ (Cu^{2+})
	961.46	5.87			Cu 2$p_{1/2}$ (Cu^{2+})

Table S4. FTIR chemical assignments

Chemical Assignment	Wavenumber, cm^{-1}								Ref.
	TA-type	TA-type +Cu	TA-type Aft.*	A-type	S-type	C-type	C-type + Cu	C-type Aft.*	
$–NH_2$ υas	3468	3510					3489		[1, 5]
$–NH_2$ υs	3391	3428							[1, 5]
>NH υ, –OH υs (Overlaped)	3436	3453	3445	3432	3445	3436	3416	3454	[5-7]
C-H υas	2923	2927	2944	2923	2936	2927	2931	2932	[1, 8]
C-H υs	2858	2851	2865	2853	2869	2857	2858	2868	[5, 8]
CO_2 H-bonding	2422, 2391	2365, 2344	2439, 2402	2357, 2334	2365, 2336	2435, 2356	2427, 2373	2433, 2407	[9]
C=O υs (Amide I), and (1°/2°) amine bend. overlapping	1602	1609	1605	1602	1609	1609	1610	1605	[7, 8]
N-H bend. (Amide II), (primary amine), C-H and (1°/2°) OH bend. and COO^- salt	1411	1412	1412	1408	1412	1404	1400	1412	[8, 10]
C-N υ, CH_2 sym. def., -C-O str. (1° –OH υs)	1388	1388	1388	1388	1387	1392	1387	1388	[2, 8]
C-N stretching	1330 - 1261	1330 - 1256	1339-1277	1330 - 1265	1339 - 1281	1330 - 1265	1329 - 1261	1334 - 1264	[2, 11]
C-O-C bridge υs	1117	1113	1121	1113	1117	1113	1112	1116	[8]
C-O υs, primary OH groups, carbohydrate ring	932	936	936	928	933	932	936	932	[5, 8]
C-H out-of-plane bend.	813	854	854	846	798	855	853	858	[11]
-NH twist	714	714	723	710	719	722	714	718	[1, 5]
Fe-O-Fe υs and C-H bending	616	624	624	616	624	627	624	620	[1, 6, 12]
$υ1$ of the bulk Fe_3O_4 (Fe_{tetra}–O)	541	554	558	558		591	541	558	[11, 12]
bulk magnetite's $υ2$ (Fe_{Octa}–O bond)	472	475	476	473	480	480	456	467	[4, 10]

* TA-type Aft. 6th regeneration cycles

Table S5. Adsorption kinetics parameters of Cu(II) adsorption.

Sorbent	$q_{m., exp.*}$	PFORE			PSORE			Intraparticle diffusion		
		K_1, min^{-1}	qe,*	R^2	K_2, min^{-1}	$q_{e,2}$, *	R^2	$K_{id,1}$,**	$K_{id,2}$,**	$K_{id,3}$,**
r-MCS	0.40191357	0.01681	0.273	0.9515	193.537088	0.41955108	0.9939	0.0356	0.0160	0.0015
TA-type	1.57932836	0.08268	1.183	0.9231	0.50392365	1.60745861	0.9994	0.3456	213.0000	0.0002
A-type	0.71916407	0.05182	0.470	0.9643	8.48194967	0.75528701	0.9992	0.1116	0.0192	0.0023
S-type	0.81484279	0.05435	0.576	0.9481	7.10973499	0.850051	0.9989	0.1409	0.0611	0.0026
C-type	0.91650143	0.08314	0.866	0.9758	5.11265545	0.95556617	0.9986	0.162	0.063	0..14

Units: *: mmol Cu. g^{-1}, ** : mg g^{-1} $min^{-0.5}$

Table S6. Adsorption isotherms parameters of Cu(II) adsorption.

Sorbent	T, K	$q_{m.\ Exp}$[a]	Langmuir isotherm			Freundlich isotherm			Temkin isotherm		
			$q_{m.L,}$[a]	b_L[b]	R^2	1/n	k_f,[a]	R^2	A_T[c]	B_T[d]	R^2
r-MCS	298	0.8614	1.0349	0.9877	0.9908	0.4382	0.4598	0.9699	10.5941	0.2184	0.9552
	308	0.9067	1.0735	1.0728	0.9911	0.4188	0.4970	0.9705	11.9819	0.2228	0.9665
	318	0.9448	1.1046	1.1828	0.9935	0.4064	0.5323	0.9694	13.4086	0.2274	0.9660
	328	0.9845	1.1305	1.3639	0.9962	0.3905	0.5753	0.9678	15.7841	0.2302	0.9721
TA-type	298	3.2647	3.4258	4.7696	0.9973	0.3031	2.4138	0.9613	148.2945	0.5161	0.9866
	308	3.1703	3.3411	4.1512	0.9969	0.3116	2.2877	0.9752	120.2412	0.5135	0.9789
	318	3.0507	3.2352	3.7834	0.9967	0.3146	2.1727	0.9794	106.1868	0.5024	0.9768
	328	2.9456	3.1397	3.2566	0.9958	0.3213	2.0389	0.9843	87.0167	0.4954	0.9693
A-type	298	1.4755	1.7609	1.1342	0.9962	0.4890	0.8006	0.9380	11.1496	0.3848	0.9768
	308	1.5441	1.8038	1.3257	0.9975	0.4621	0.8768	0.9333	13.3378	0.3876	0.9788
	318	1.6344	1.8660	1.5592	0.9988	0.4354	0.9669	0.9263	16.4364	0.3911	0.9832
	328	1.6974	1.9124	1.7594	0.9990	0.4219	1.0342	0.9102	18.9993	0.3957	0.9807
S-type	298	1.5438	1.7784	1.4537	0.9980	0.4430	0.8978	0.9356	11.1496	0.3848	0.9768
	308	1.5998	1.8272	1.5878	0.9982	0.4373	0.9519	0.9145	13.3378	0.3876	0.9788
	318	1.6671	1.8797	1.7955	0.9990	0.4153	0.9757	0.9165	16.4364	0.3911	0.9832
	328	1.7348	1.9268	2.0417	0.9993	0.3985	1.0965	0.9059	18.9993	0.3957	0.9807
C-type	298	1.7030	1.9361	1.6418	0.9984	0.4277	1.0221	0.9342	14.9259	0.3772	0.9792
	308	1.7660	1.9865	1.8213	0.9970	0.4184	1.0857	0.9216	16.4644	0.3854	0.9752
	318	*1.8550*	2.0708	1.9718	0.9987	0.4090	1.1620	0.9159	19.6446	0.3867	0.9771
	328	1.9133	2.1277	2.0880	0.9989	0.4048	1.2154	0.9067	23.3244	0.3885	0.9767

Units: [a]: mmol $Cu.g^{-1}$, [b]: L $mmol^{-1}$, [c]A_T: L $mmol^{-1}$, and [d]B_T: kJ mol^{-1}.

Table S7. Thermodynamic parameters for Cu(II) adsorption.

Sorbent	Temp., (K)	ΔH°, (KJ mol^{-1})	ΔS°, (J mol^{-1})	ΔG°, (KJ mol^{-1})	TΔS°, (KJ mol^{-1})	R^2
r-MCS	298	8.623	119.52	-26.99	35.62	0.9759
	308			-28.19	36.81	
	318			-29.38	38.01	
	328			-30.58	39.20	
TA-type	298	-10.045	70.12	-30.94	20.89	0.9909
	308			-31.64	21.60	
	318			-32.34	22.30	
	328			-33.04	23.00	
A-type	298	12.033	132.26	-27.38	39.41	0.9983
	308			-28.70	40.74	
	318			-30.03	42.06	
	328			-31.35	43.38	
S-type	298	9.253	124.86	-27.96	37.21	0.9878
	308			-29.20	38.46	
	318			-30.45	39.71	
	328			-31.70	40.95	
C-type	298	6.527	116.92	-28.32	34.84	0.9899
	308			-29.48	36.01	
	318			-30.65	37.18	
	328			-31.82	38.35	

Table S8. Metal desorption and adsorption cycles.

Cycles	r-MCS		TA-type		A-type		S-type		C-type	
	**SE, %**	**DE, (%)**	**SE, %**	**DE, (%)**	**SE, %**	**DE, (%)**	**SE, %**	**DE, (%)**	**SE, %**	**DE, (%)**
I	100.0*	98.69	100.0*	98.95	100.0*	99.04	100.0*	99.14	100.0*	99.09
II	97.69	96.89	98.98	98.19	98.12	98.01	98.55	98.06	98.45	98.35
III	96.42	95.26	98.13	97.48	94.84	97.18	93.72	97.23	97.35	97.93
IV	93.79	93.11	97.11	97.01	93.26	95.25	92.66	96.31	95.00	95.64
V	91.48	90.96	95.97	96.58	92.43	94.35	91.72	95.88	93.55	94.83
VI	89.25	90.13	94.84	94.45	91.64	93.81	90.98	94.13	92.40	94.42
**Δ($1^{st}/6^{th}$)**	**10.75**	**8.56**	**5.16**	**4.49**	**8.36**	**5.23**	**9.02**	**5.00**	**7.60**	**4.67**

*Standard metal ion sorption efficiency (at first cycle). **Adsorption experiment:** (C_0: 1.0 mmoL.L^{-1}; SD: 0.5 g.L^{-1}; pH_0: 5, Time: 2h, T: 298 K). **Desorption experiment:** (acidified thiourea: 0.25 mol.L^{-1} at pH: 2.0; SD: 1.0 g.L^{-1}; Time: 1h; T: 298 K)

Table S9. Chemical properties of selected metal ions.

Element	At. No.	Hydrated Species	M-O bond Distance (Å)	M^{n+} ion radius (Å)	M^{n+} ion radius, (Å)[b]	Effective ionic charge	Electronegativity (Pauling units)	qm, mmol metal g^{-1}				
								r-MCS	TA-type	A-type	S-type	C-type
Co	27	$Co(H_2O)_6^{2+}$	2.08	0.74	0.745	12.95	1.88	0.23	0.56	0.32	0.39	0.08
Ni	28	$Ni(H_2O)_6^{2+}$	2.055	0.715	0.69	13.6	1.91	0.18	0.48	0.26	0.24	0.23
Cu	29	$Cu(H_2O)_6^{2+}$	2.14	0.73	0.6	14.25	1.9	0.58	1.73	1.07	1.10	1.29
Zn	30	$Zn(H_2O)_6^{2+}$	2.08	0.74	0.74	14.9	1.65	0.13	0.41	0.19	0.30	0.21
Cd	48	$Cd(H_2O)_6^{2+}$	2.3	0.96	0.95	16.4	1.69	0.06	0.15	0.10	0.09	0.26

Table S10. The parameters and constants of Generalized Langmuir equation and Goodness of fit for all adsorbents.

$$q_{(pH_0, C_0)} = \frac{q_m \times k \times \mathrm{pH}^m \times C^n}{(1 + k \times pH^f \times C^e)^g}$$

Parameters	Adsorbent				
	r-MCS	TA-type	A-type	S-type	C-type
q_m, mmol g^{-1}	0.860	3.290	1.480	1.550	1.711
k	0.8628	4.1660	0.9908	1.2700	1.4340
m	5.0550	2.6150	33.7500	5.8270	5.7330
n	1.6790	3.8500	14.5800	4.6990	4.8320
f	1.2100	0.5000	2.3870	1.1000	1.2100
e	0.3047	1.3250	1.0000	0.9622	1.1000
g	4.5000	2.7140	14.2900	4.7950	4.2470
ΔG^o, kJ mol^{-1}	-27.0351	-30.9361	-27.3779	-27.9929	-28.2938
R^2	0.9812	0.9854	0.9930	0.9911	0.9905
Adj. R^2	0.9700	0.9766	0.9884	0.9852	0.9829
SSE $\times 10^{-3}$	8.066	133.4	12.71	21.01	22.15

Table 11. Comparison for experimental adsorption capacity ($q_{e,exp}$) and mathematically calculated ($q_{e,cal.}$) for r-MCS, TA-type, A-type, S-type, and C-type adsorbents.

Factor	pH_0	r-MCS			TA-type			A-type			S-type			C-type		
		$q_{e,exp.}$	$q_{e,cal}$	Error, %	$q_{e,exp.}$*	$q_{e,cal.}$**	Error, %	$q_{e,exp}$*	$q_{e,cal.}$**	Error, %	$q_{e,exp.}$	$q_{e,cal}$	Error, %	$q_{e,exp.}$	$q_{e,cal}$	Error, %
pH_0	2.09	0.07	0.11	-58.24	0.28	0.47	-71.01	0.13	0.13	2.64	0.17	0.22	-30.59	0.21	0.28	-33.33
	3.08	0.19	0.29	-48.66	1.04	0.99	4.61	0.43	0.43	0.28	0.49	0.47	5.41	0.56	0.54	4.69
	4.08	0.31	0.32	-4.02	1.44	1.39	3.65	0.6	0.61	-2.35	0.66	0.65	1.81	0.79	0.76	3.95
	5.03	0.4	0.39	2.36	1.58	1.66	-4.96	0.72	0.7	3.25	0.81	0.84	-3.89	0.91	0.93	-2.22
	6.06	0.4	0.41	-3.45	1.57	1.67	-6.27	0.72	0.72	0.25	0.82	0.86	-5.42	0.92	0.9	2.37
	7.01	0.41	0.42	-1.84	1.56	2.61	-66.53	0.71	0.72	-1.75	0.83	1.2	-44.51	0.93	1.28	-38.15

Factor	C_0, mmol $.L^{-1}$	r-MCS			TA-type			A-type			S-type			C-type		
		$q_{e,exp.}$	$q_{e,cal}$	Error, %	$q_{e,exp.}$*	$q_{e,cal.}$**	Error, %	$q_{e,exp.}$*	$q_{e,calc.}$**	Error, %	$q_{e,exp.}$	$q_{e,calc}$	Error, %	$q_{e,exp.}$	$q_{e,calc}$	Error, %
C0, mmol $Cu.L^{-1}$	0.32	0.23	0.2105	8.48	0.6	0.55	7.78	0.2845	0.2657	6.61	0.3242	0.2957	8.79	0.361	0.325	9.97
	0.65	0.32	0.3114	2.69	1.18	1.23	-4.2	0.4951	0.5021	-1.41	0.5722	0.616	-7.65	0.6288	0.6779	-7.81
	1	0.39	0.3851	1.26	1.57	1.63	-2.84	0.7165	0.6975	2.65	0.8043	0.8231	-2.34	0.9057	0.9135	-0.86
	1.58	0.55	0.5047	8.24	2.15	2.09	2.78	0.9905	0.9417	4.93	1.0578	1.0689	-1.05	1.1661	1.1643	0.15
	2.37	0.71	0.6403	9.82	2.62	2.47	5.86	1.2142	1.1567	4.74	1.2961	1.2523	3.38	1.4487	1.3551	6.46
	3.18	0.79	0.7113	9.96	2.99	2.81	6.12	1.3625	1.2865	5.58	1.4519	1.383	4.75	1.5966	1.496	6.3
	4.73	0.86	0.7996	7.02	3.26	3.11	4.53	1.4755	1.3964	5.36	1.5438	1.5148	1.88	1.703	1.6495	3.14
	6.27	0.86	0.9025	-4.94	3.29	3.41	-3.51	1.4802	1.5144	-2.31	1.5494	1.6098	-3.9	1.7109	1.7667	-3.26

* $q_{e, exp.}$: qe experimentally and ** $q_{e,cal.}$, calculated qe mathematically from the equation. Both unit: in mmol $Cu.g^{-1}$

List of Figures

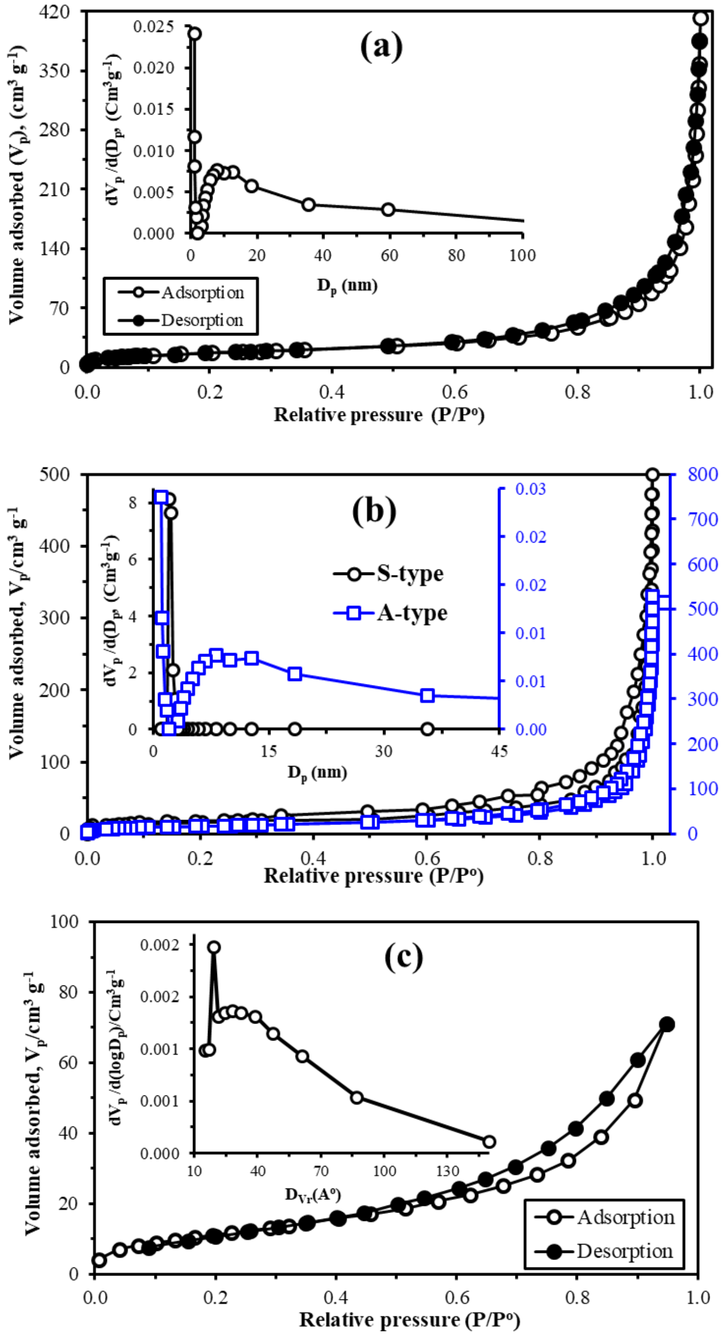

Figure S1. Textural characterization of TA-type (a), S-type, A-type (b), and C-type (c): BET surface area analysis and Pore size analysis (insert).

Parameter	TA-type	S-type	A-type	C-type
SSA ($m^2 g^{-1}$)	75.27	61. 052	60. 382	42.56
V_P (cm^3 STP g^{-1})	0.5696	0.4105	0.3598	0.1134
Mean pore diameter (nm)	27.533	26.997	22.466	3.839

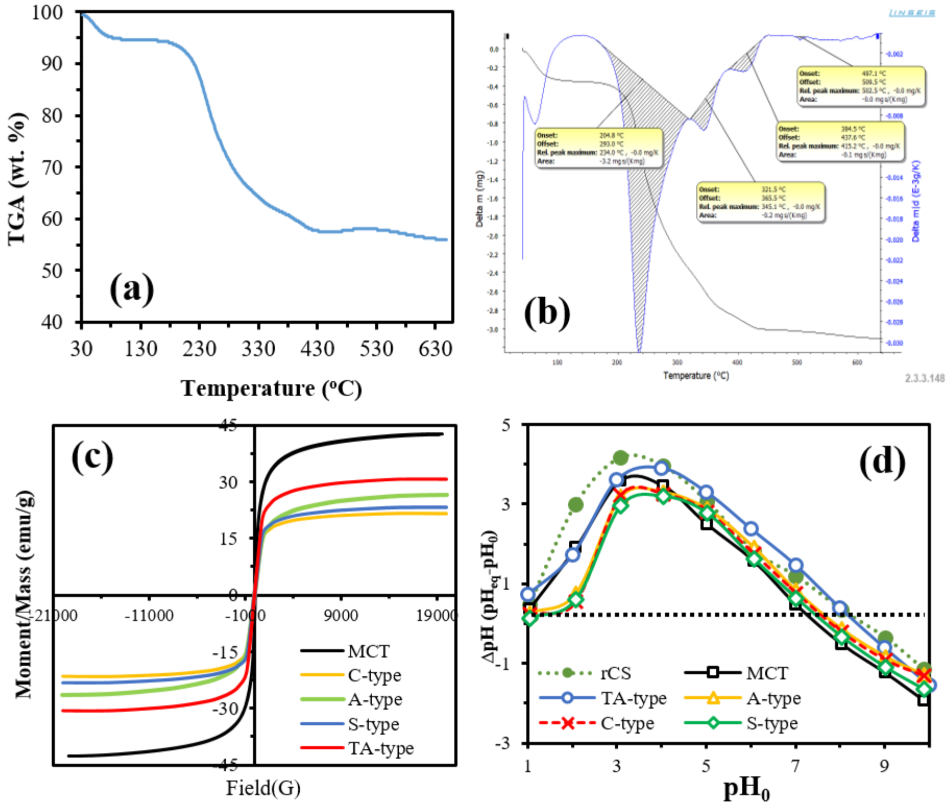

Figure S2. Thermogravimetric analysis of S-type nanohybrid: TGA (**a**) and DTG (**b**), magnetization curves (**c**) and the pHZPC of rCS, MCS, and all nanohybrids (**d**).

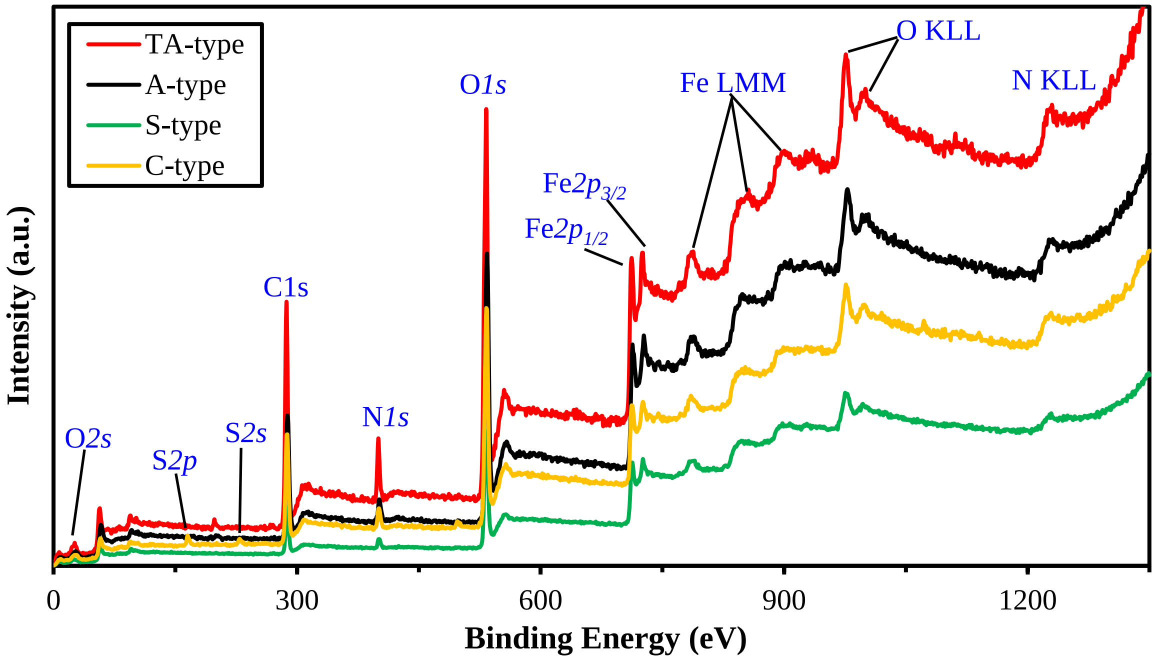

Figure S3. XPS survey spectra of TA-type, A-type, S-type and C-type nanocomposites.

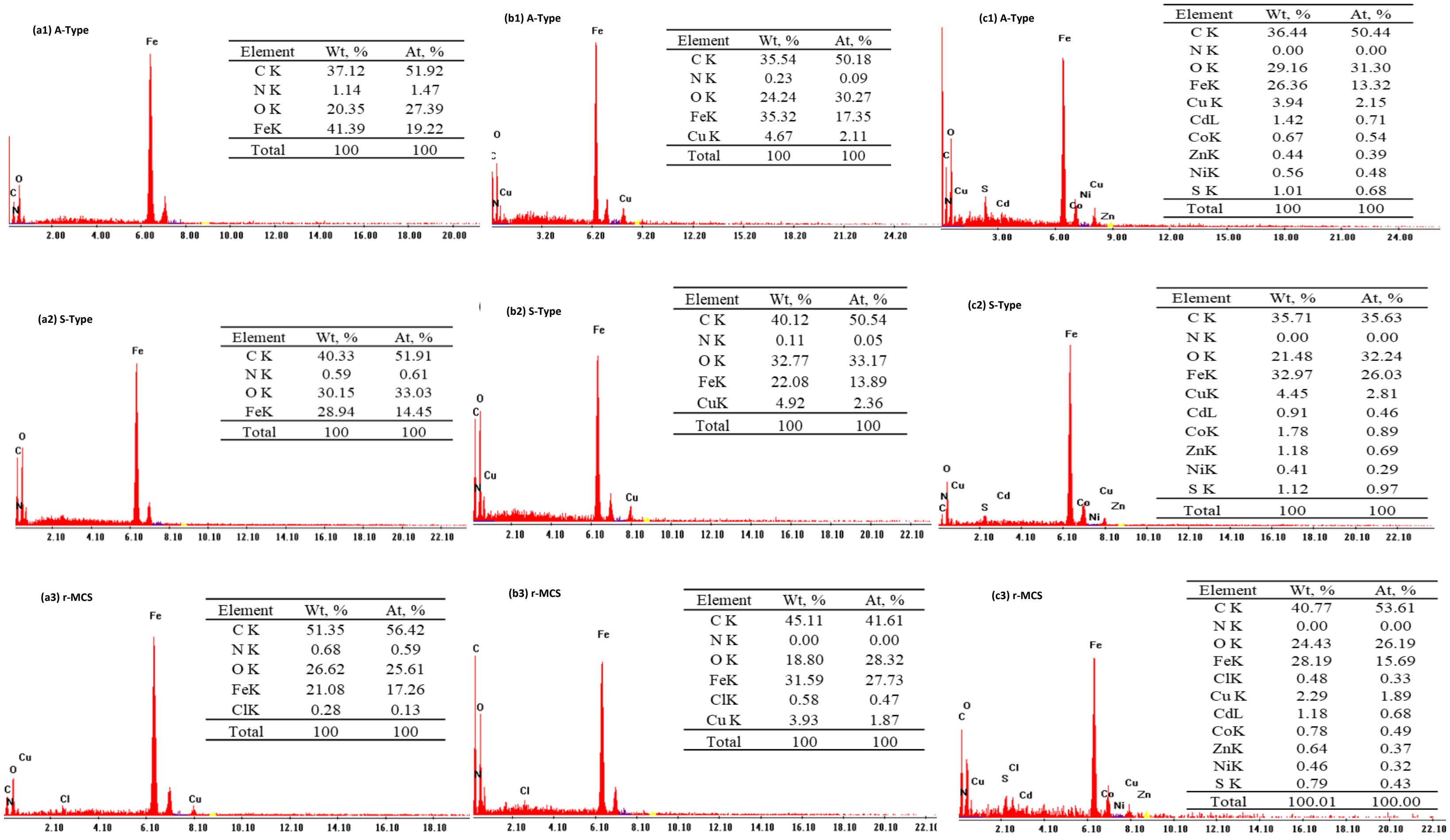

(a1) A-Type

Element	Wt, %	At, %
C K	37.12	51.92
N K	1.14	1.47
O K	20.35	27.39
FeK	41.39	19.22
Total	100	100

(b1) A-Type

Element	Wt, %	At, %
C K	35.54	50.18
N K	0.23	0.09
O K	24.24	30.27
FeK	35.32	17.35
Cu K	4.67	2.11
Total	100	100

(c1) A-Type

Element	Wt, %	At, %
C K	36.44	50.44
N K	0.00	0.00
O K	29.16	31.30
FeK	26.36	13.32
Cu K	3.94	2.15
CdL	1.42	0.71
CoK	0.67	0.54
ZnK	0.44	0.39
NiK	0.56	0.48
S K	1.01	0.68
Total	100	100

(a2) S-Type

Element	Wt, %	At, %
C K	40.33	51.91
N K	0.59	0.61
O K	30.15	33.03
FeK	28.94	14.45
Total	100	100

(b2) S-Type

Element	Wt, %	At, %
C K	40.12	50.54
N K	0.11	0.05
O K	32.77	33.17
FeK	22.08	13.89
CuK	4.92	2.36
Total	100	100

(c2) S-Type

Element	Wt, %	At, %
C K	35.71	35.63
N K	0.00	0.00
O K	21.48	32.24
FeK	32.97	26.03
CuK	4.45	2.81
CdL	0.91	0.46
CoK	1.78	0.89
ZnK	1.18	0.69
NiK	0.41	0.29
S K	1.12	0.97
Total	100	100

(a3) r-MCS

Element	Wt, %	At, %
C K	51.35	56.42
N K	0.68	0.59
O K	26.62	25.61
FeK	21.08	17.26
ClK	0.28	0.13
Total	100	100

(b3) r-MCS

Element	Wt, %	At, %
C K	45.11	41.61
N K	0.00	0.00
O K	18.80	28.32
FeK	31.59	27.73
ClK	0.58	0.47
Cu K	3.93	1.87
Total	100	100

(c3) r-MCS

Element	Wt, %	At, %
C K	40.77	53.61
N K	0.00	0.00
O K	24.43	26.19
FeK	28.19	15.69
ClK	0.48	0.33
Cu K	2.29	1.89
CdL	1.18	0.68
CoK	0.78	0.49
ZnK	0.64	0.37
NiK	0.46	0.32
S K	0.79	0.43
Total	100.01	100.00

Figure S4. EDX measurements of raw r-MCS, A-type, and S-type materials before (a), and after Cu(II) adsorption from synthetic solution (b), and mixed system(c).

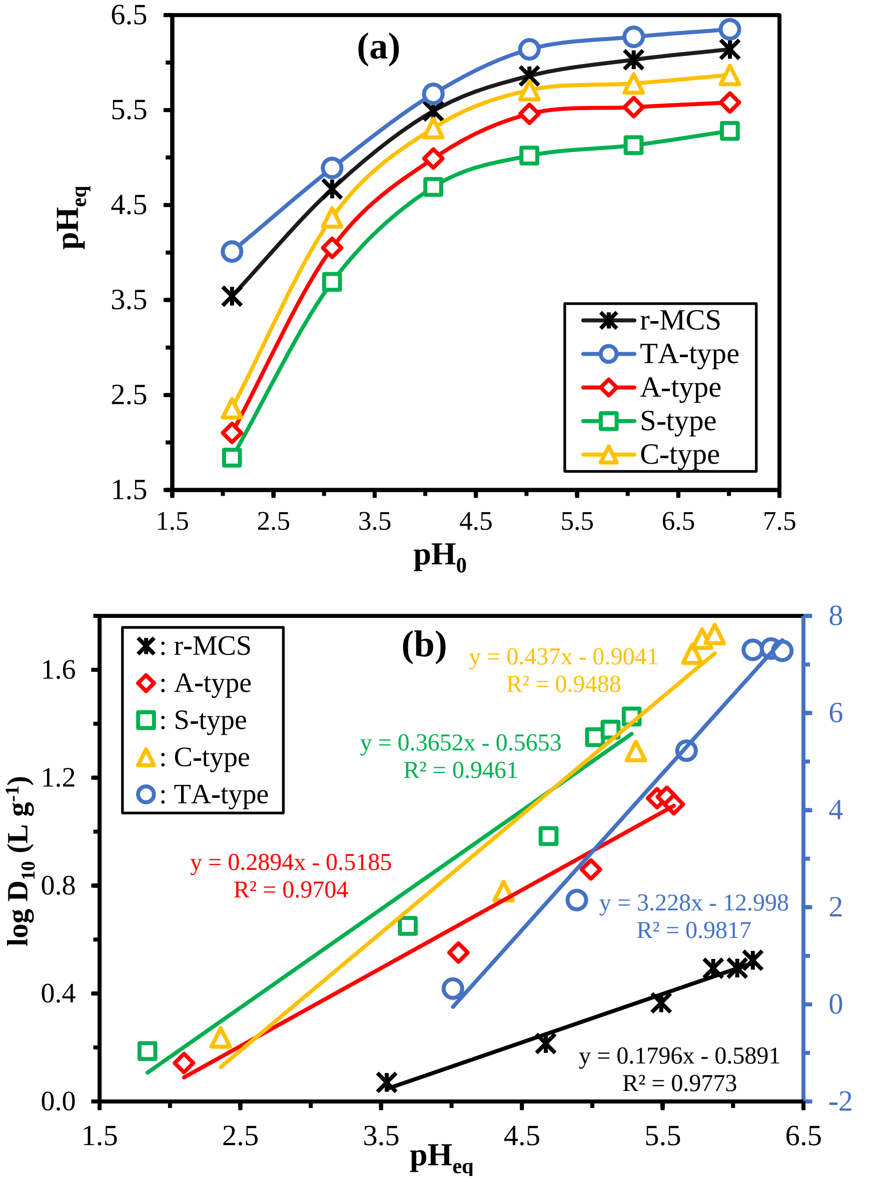

Figure S5. Effect of initial pH versus equilibrium pH (**a**) and Plot of distribution ratio (D, L g^{-1}) vs. pHeq (**b**). (C_0: 1.0 mmol L^{-1}; T: 25 °C; SD: 0.5 g L^{-1}; agitation time: 12 h).

.

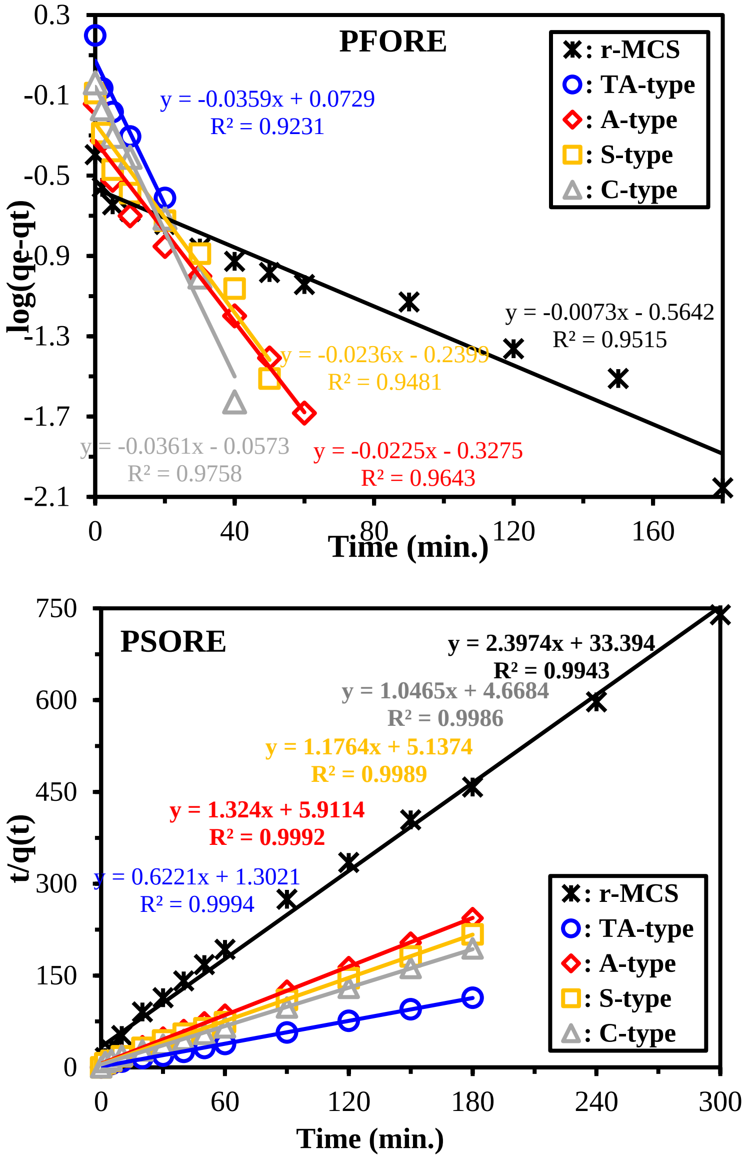

Figure S6. Kinetics models PFORE and PSORE for Cu(II) sorption. (pH_0: 5.05, C_0: 1.0 mmol Cu L^{-1}, SD: 0.5 g L^{-1}, Temp.: 298 K, agitation speed: 150 rpm).

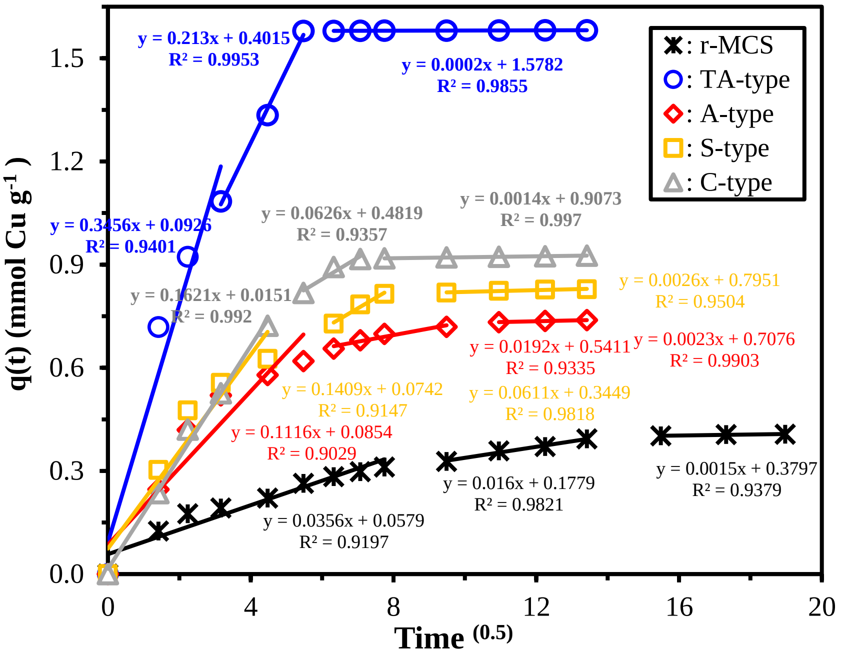

Figure S7. Kinetics models of interparticle diffusion for Cu(II) adsorption. (pH_0: 5.05, C_0: 1.0 mmol Cu L^{-1}, SD: 0.5 g L^{-1}, Temp.: 298 K, agitation speed: 150 rpm).

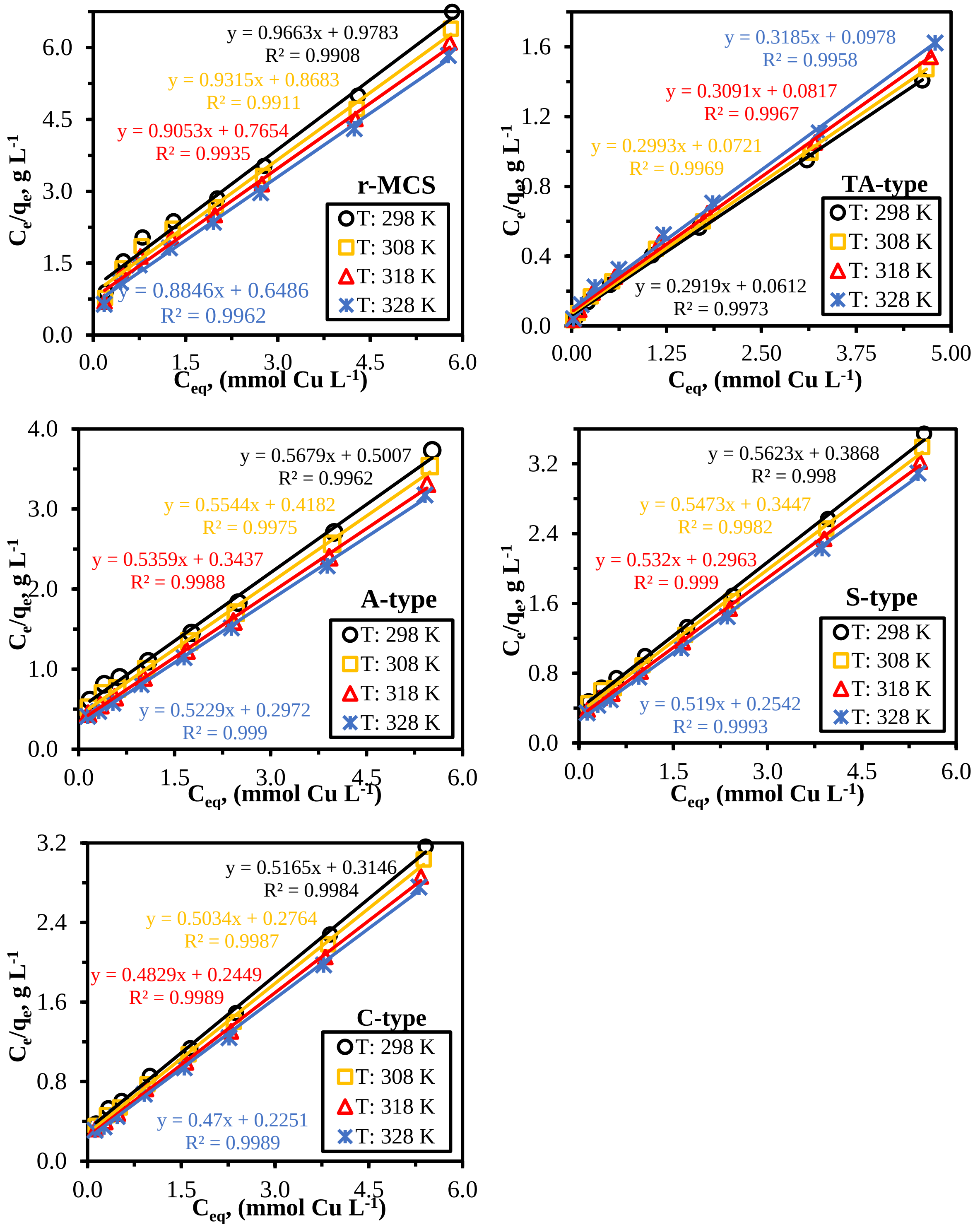

Figure S8a. Langmuir Isotherm models for Cu(II) sorption. (pH_0: 5.0, C_0: 0.067-1.467 mmol Cu L^{-1}, SD: 0.5 g L^{-1}, Temp.:298-328 K, agitation speed: 150 rpm).

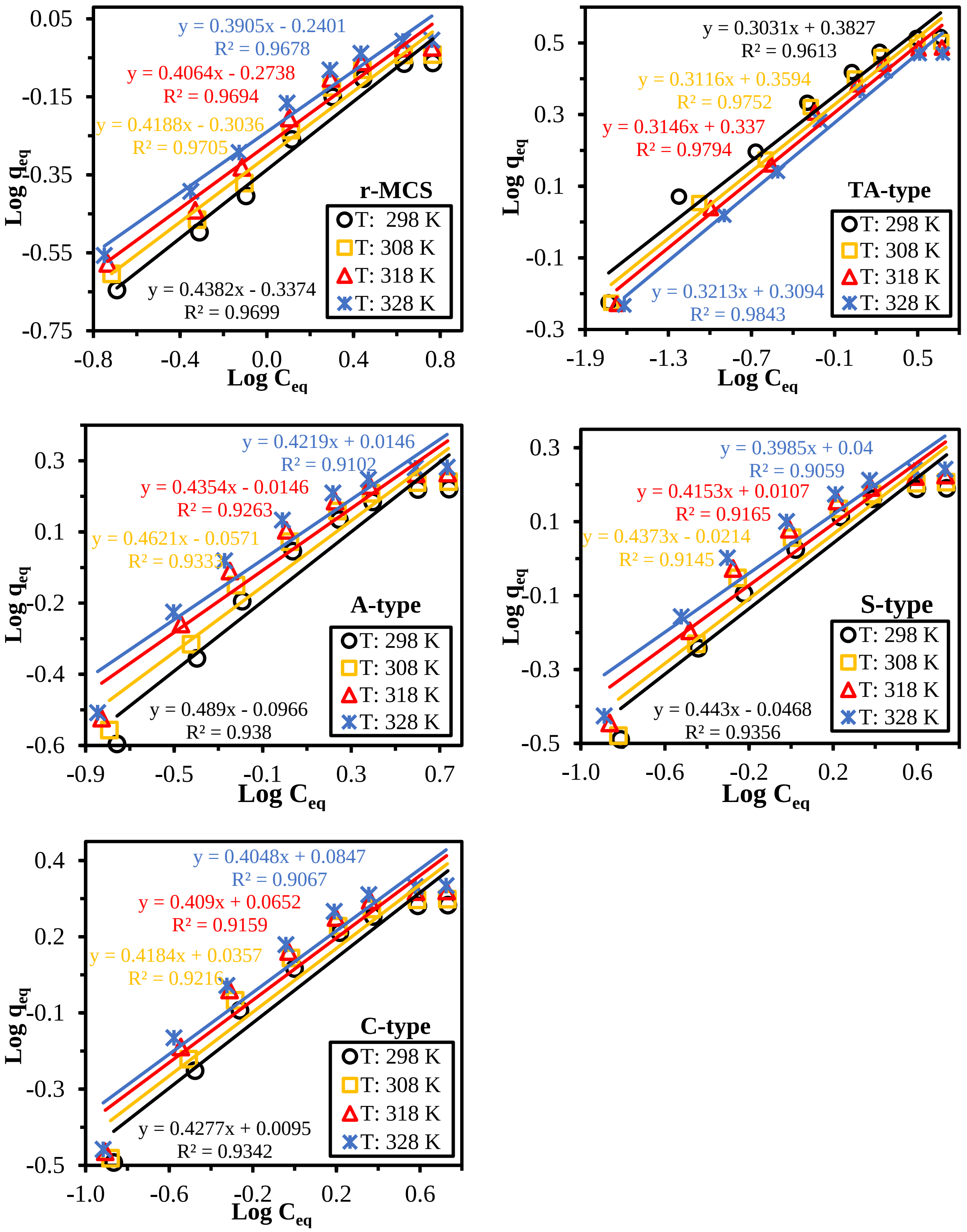

Figure S8b. Freundlich isotherm models for Cu(II) sorption. (pH_0: 5.0, C_0: 0.067-1.467 mmol Cu L^{-1}, SD: 0.5 g L^{-1}, Temp.:298-328 K, agitation speed: 150 rpm).

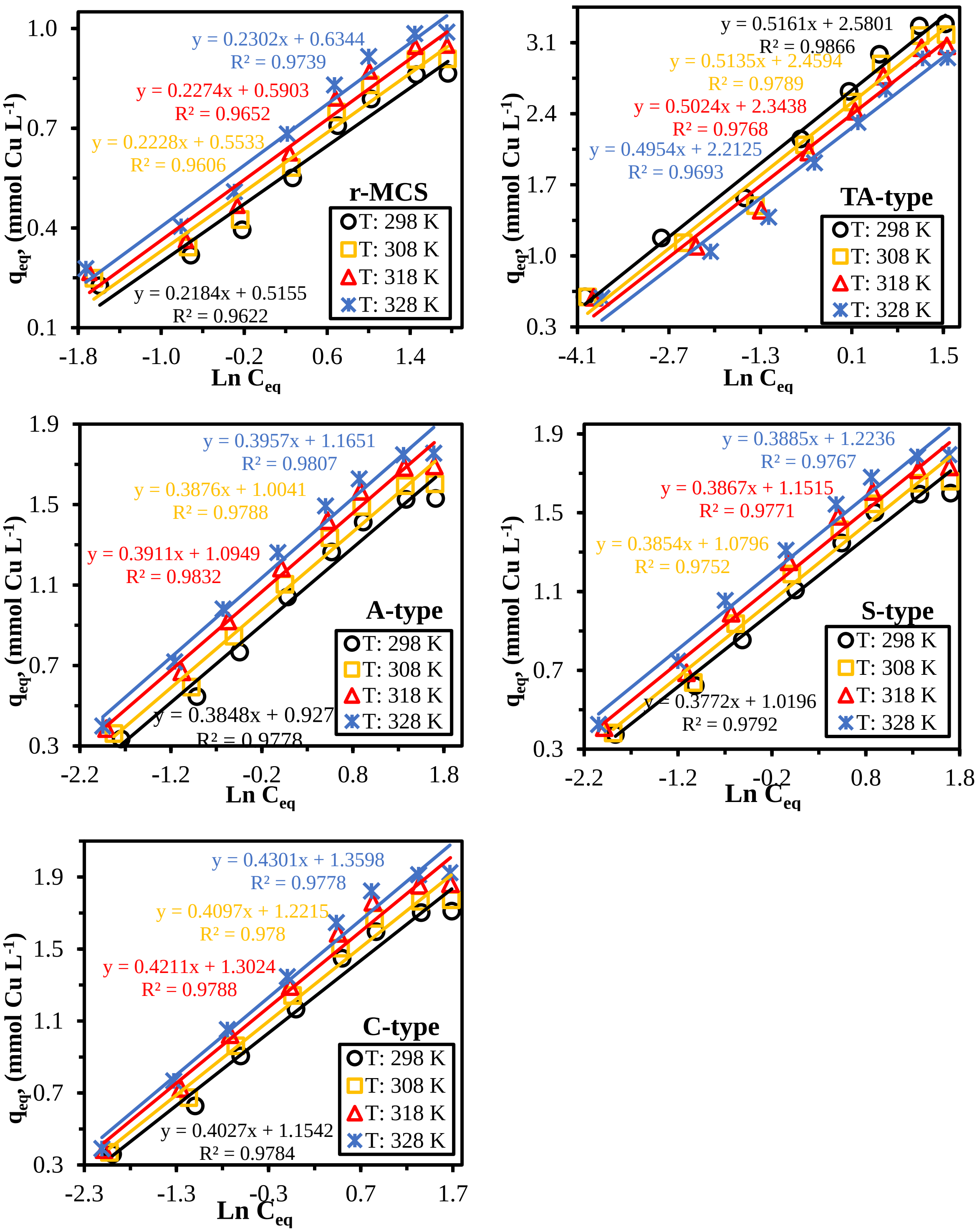

Figure S8c. Temkin isotherm models for Cu(II) sorption. (pH_0: 5.0, C_0: 0.067-1.467 mmol Cu L^{-1}, SD: 0.5 g L^{-1}, Temp.:298-328 K, agitation speed: 150 rpm).

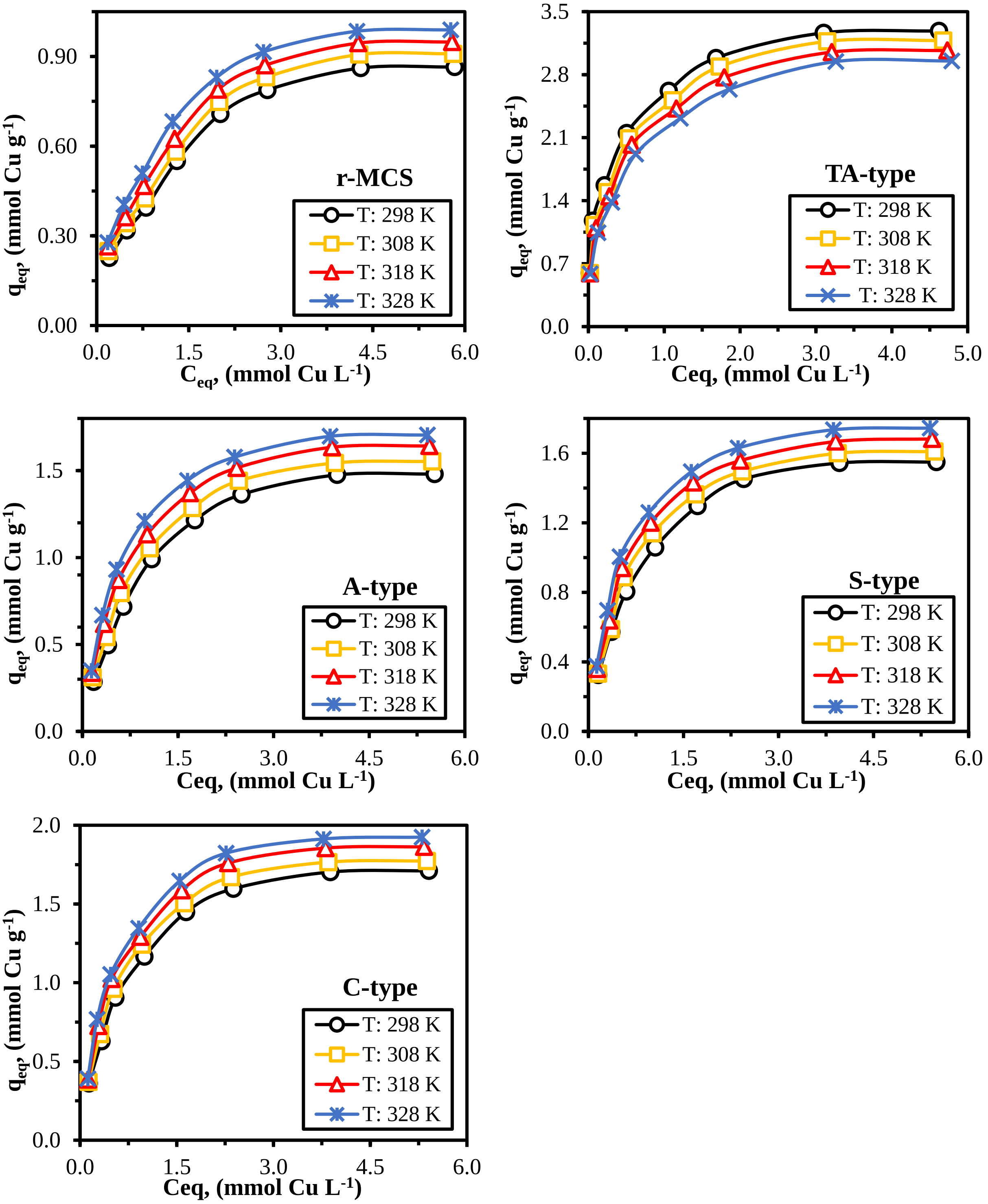

Figure S9. Sorption isotherms for all adsorbents types at different temperatures. (pH_0: 5.06; C_0: 0.317-6.265 mmol Cu L^{-1}; SD: 0.5 g L^{-1}, Temp, (i.e. T: 298, 308, 318, and 328 K), time: 120 min for TA-, A-, S-, and C-type, and 240 min for r-MCS).

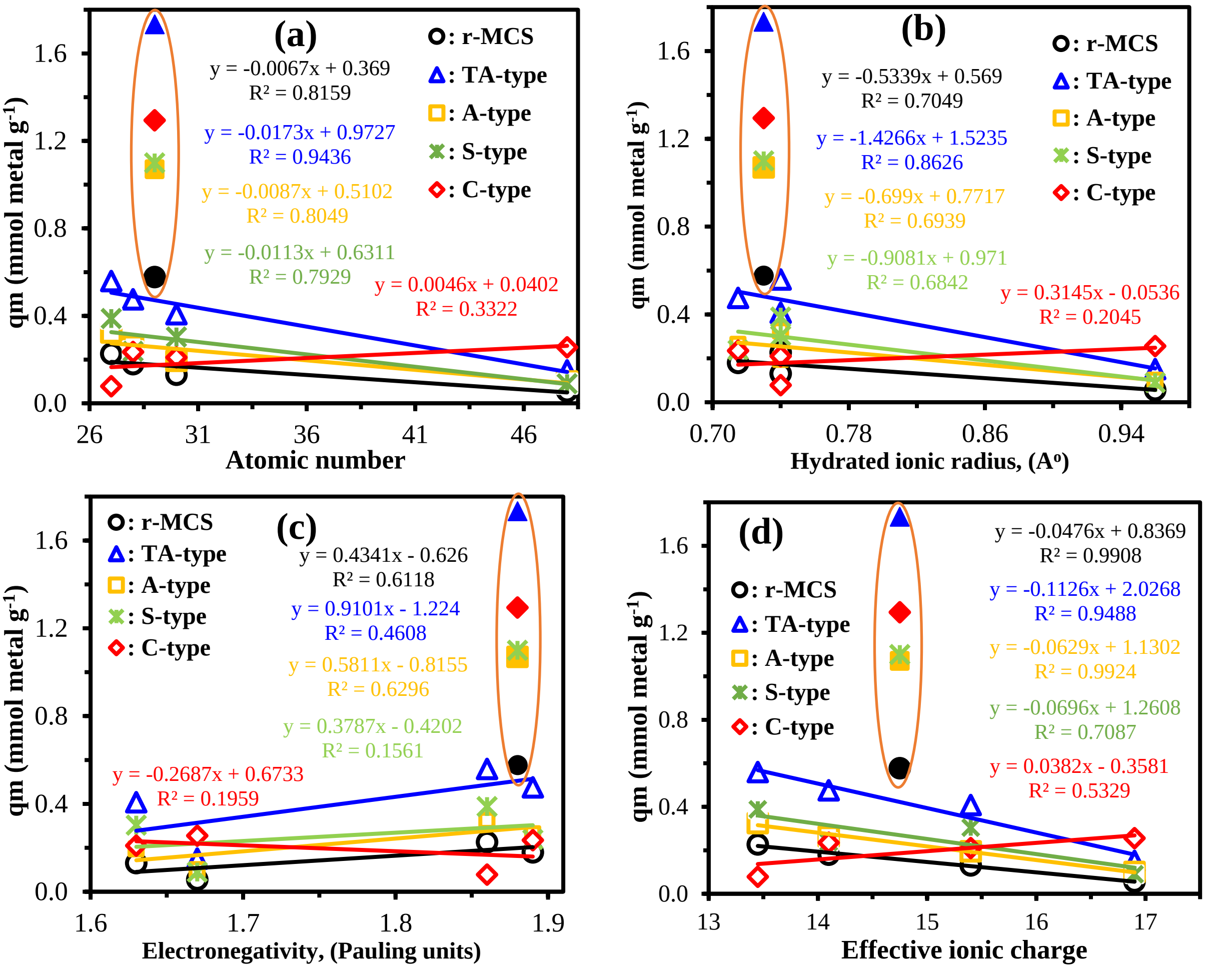

Figure S10. Correlation between intrinsic physicochemical characteristics (atomic number (a), hydrated ionic radius (b), electronegativity (c), and effective ionic charge (d)) and their sorption capacities (qm).

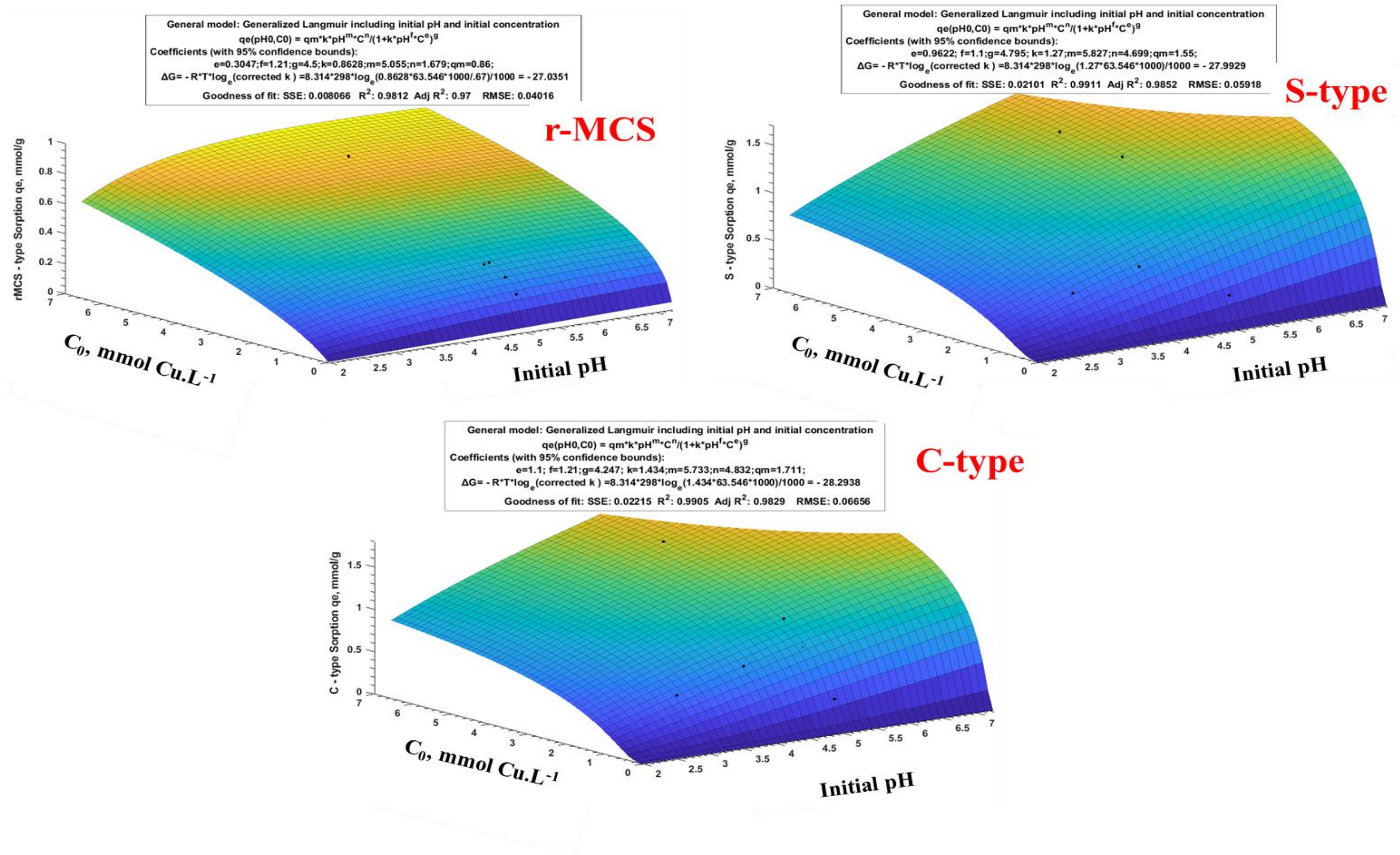

Figure S11. Generalized Langmuir including initial pH and initial Cu(II) concentration.

Reference

[1] A. Benettayeb, A. Morsli, K.Z. Elwakeel, M.F. Hamza, E. Guibal, Recovery of heavy metal ions using magnetic glycine-modified chitosan—Application to aqueous solutions and tailing leachate, Appl. Sci. 11 (2021) 8377.
[2] A.A. Galhoum, T. Akashi, M. Linnolahti, J.T. Hirvi, A.G. Al-Sehemid, A. Kalam, E. Guibal, Functionalization of poly(glycidylmethacrylate) with iminodiacetate and imino phosphonate groups for enhanced sorption of neodymium - sorption performance and molecular modeling, React. Funct. Polym. 180 (2022) 105389.
[3] C. Tien, Adsorption calculations and modeling, Butterworth-Heinemann, Boston, 1994.
[4] A.A. Al-Ghamdi, A.A. Galhoum, A. Alshahrie, Y.A. Al-Turki, A.M. Al-Amri, S. Wageh, Mechanistic studies of uranyl interaction with functionalized mesoporous chitosan-superparamagnetic nanocomposites for selective sorption: Characterization and sorption performance, Mater. Today Commun. 33 (2022) 104536.
[5] J. Coates, Interpretation of infrared spectra: Apractical Approach. In Encyclopedia of analytical chemistry, John Wiley & Sons Ltd, Chochester2000.
[6] A.A. Galhoum, M.G. Mahfouz, N.M. Gomaa, T. Vincent, E. Guibal, Chemical modifications of chitosan nano-based magnetic particles for enhanced uranyl sorption, Hydrometallurgy 168 (2017) 127-134.
[7] S.M. Pourmortazavi, H. Sahebi, H. Zandavar, S. Mirsadeghi, Fabrication of Fe_3O_4 nanoparticles coated by extracted shrimp peels chitosan as sustainable adsorbents for removal of chromium contaminates from wastewater: The design of experiment, Compos. B. Eng. 175 (2019) 107130.
[8] S.Y. Oh, D.I. Yoo, Y. Shin, H.C. Kim, H.Y. Kim, Y.S. Chung, W.H. Park, J.H. Youk, Crystalline structure analysis of cellulose treated with sodium hydroxide and carbon dioxide by means of X-ray diffraction and FTIR spectroscopy, Carbohyd. Res. 340(15) (2005) 2376-2391.
[9] A.A. Al-Ghamdi, A.A. Galhoum, A. Alshahrie, Y.A. Al-Turki, A.M. Al-Amri, S. Wageh, Mesoporous magnetic cysteine functionalized chitosan nanocomposite for selective uranyl ions sorption: experimental, structural characterization, and mechanistic studies, Polymers 14 (2022) 2568.
[10] M. Yamaura, R.L. Camilo, L.C. Sampaio, M.A. Macêdo, M. Nakamura, H.E. Toma, Preparation and characterization of (3-aminopropyl)triethoxysilane-coated magnetite nanoparticles, J. Magn. Magn. Mater. 279 (2004) 210-217.
[11] N.A. Alghamdi, Mesoporous magnetic-polyaminated-chitosan nanocomposite for selective uranium removal: performance and mechanistic studies Int. J. Environ. Sci. Technol. (2022) https://doi.10.1007/s13762-022-04565-2
[12] M. Stoia, R. Istratie, C. Păcurariu, Investigation of magnetite nanoparticles stability in air by thermal analysis and FTIR spectroscopy, J. Therm. Anal. Calorim. 125(3) (2016) 1185-1198.
